# Metal‐Catalyzed Haloalkynylation Reactions

**DOI:** 10.1002/chem.202103046

**Published:** 2021-11-18

**Authors:** Mathis Kreuzahler, Gebhard Haberhauer

**Affiliations:** ^1^ Institut für Organische Chemie Universität Duisburg-Essen Universitätsstraße 7 45117 Essen Germany

**Keywords:** catalysis, C−C coupling, haloalkynes, haloalkynylation

## Abstract

Metal catalysis has revolutionized synthetic chemistry, leading to entirely new, very efficient transformations, which enable access to complex functionalized molecules. One such new transformation method is the haloalkynylation reaction, in which both a halogen atom and an alkynyl unit are transferred to an unsaturated carbon‐carbon bond. This minireview summarizes the development of metal‐catalyzed haloalkynylation reactions since their beginning about a decade ago. So far, arynes, alkenes and alkynes have been used as unsaturated systems and the reactivities of these systems are summarized in individual chapters of the minireview. Especially, the last few years have witnessed a rapid development due to gold‐catalyzed reactions. Here, we discuss how the choice of the catalytic system influences the regio‐ and stereoselectivity of the addition.

## Graphical abstract

**Metal catalysts** can be used to react haloacetylenes with unsaturated compounds such as arynes, alkenes and alkynes, whereby the alkynyl unit and the halogen atom are usually added to adjacent positions. Cu, Pd and Au metal complexes have so far proved to be particularly suitable catalysts. The choice of catalyst system decisively determines the regio‐ and stereochemistry of the resulting halogenated product.

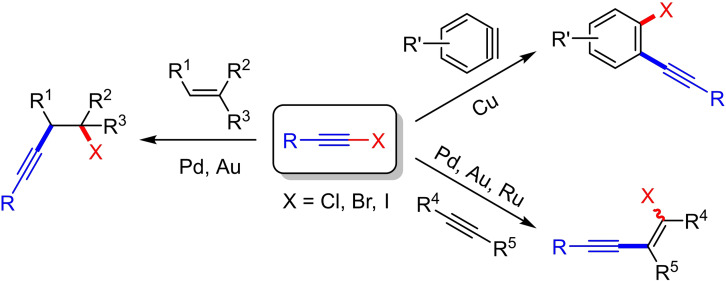

## 1 Introduction

The haloalkynylation reaction has been known for about a decade and has developed very rapidly in recent years in terms of the range of applications and the type of metal catalyst. This minireview summarizes the development of metal‐catalyzed haloalkynylation reactions that have been reported since the first publication.^[1]^ The haloalkynylation reaction is defined as the direct cleavage of an alkynyl‐halogen bond[^2^, ^3^, ^4^] followed by the reconnection of both the alkynyl and halogen unit with two carbon atoms of an unsaturated carbon‐carbon bond. The charm of this reaction type lies in the fact that, in addition to a C−C coupling reaction, another reactive group (the halogen atom) is added. This provides ready access to highly functionalized products. In typical metal‐catalyzed C−C coupling reactions the C−C bond formation generally takes place through the elimination of a reactive group. The absence of such a leaving group makes the haloalkynylation reaction atom‐economical. However, this advantage also represents the challenge of the reaction: finding a catalyst and conditions that prevent further reactions of the active groups (alkyne and halogen atom). The haloalkynylation reaction also enables access to functionalized systems that can otherwise only be obtained through several synthetic steps. In particular, the introduced halogen atom enables, depending on the type of C−X bond (C(sp^3^)‐X or (C(sp^2^)‐X), a subsequent functionalization by a nucleophilic substitution reaction or coupling reaction.

**Scheme 1:**
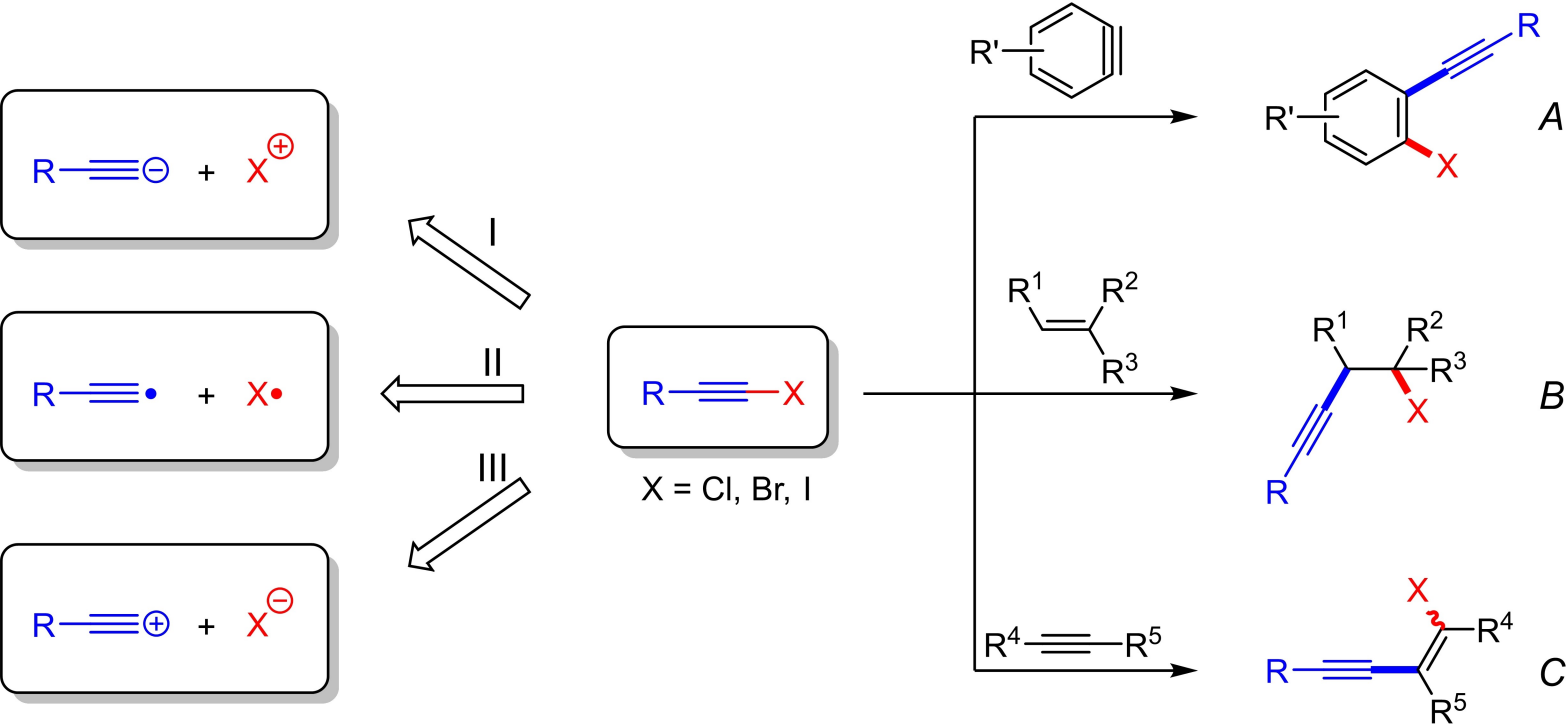
The haloalkynylation reaction of arynes (*A*), alkenes (*B*) and alkynes (*C*) can be described by means of the synthons I, II and III.

In principle, the haloalkynylation reaction can be described via three different synthons (Scheme 1), two of which are ionic and one is radical type. The transition metal and halogen atom (X=Cl, Br, I)^[5]^ of the haloalkyne^[4]^ have a significant influence on the type of synthon. In many of the examples that we will show, it is difficult to classify which synthon describes the reaction. However, attempts should be made as far as possible to assign the reactions to the corresponding synthon in order to explain and predict the regio‐ and stereoselectivity. It must be noted that the synthons do not occur as real intermediates in the reaction due to more complex rearrangement processes.

The unsaturated systems investigated so far, which can undergo a haloalkynylation reaction, include arynes (*A*), alkenes (*B*) and alkynes (*C*) (Scheme 1). In this minireview we show which metals were used for the individual unsaturated systems and how these affect the type of product. While only copper and palladium catalysts were used in the early years, there is currently a trend towards the use of cationic Au(I) complexes as catalysts. Recently, also Ru complexes have been applied. In addition, the reaction mechanisms will be used to explain how the regio‐ and stereoselectivity can be controlled by the choice of the metal catalyst.

## 2 Haloalkynylation Reactions of Arynes (Direct Synthesis of *ortho*‐Alkynylaryl Halides)

**Scheme 2:**
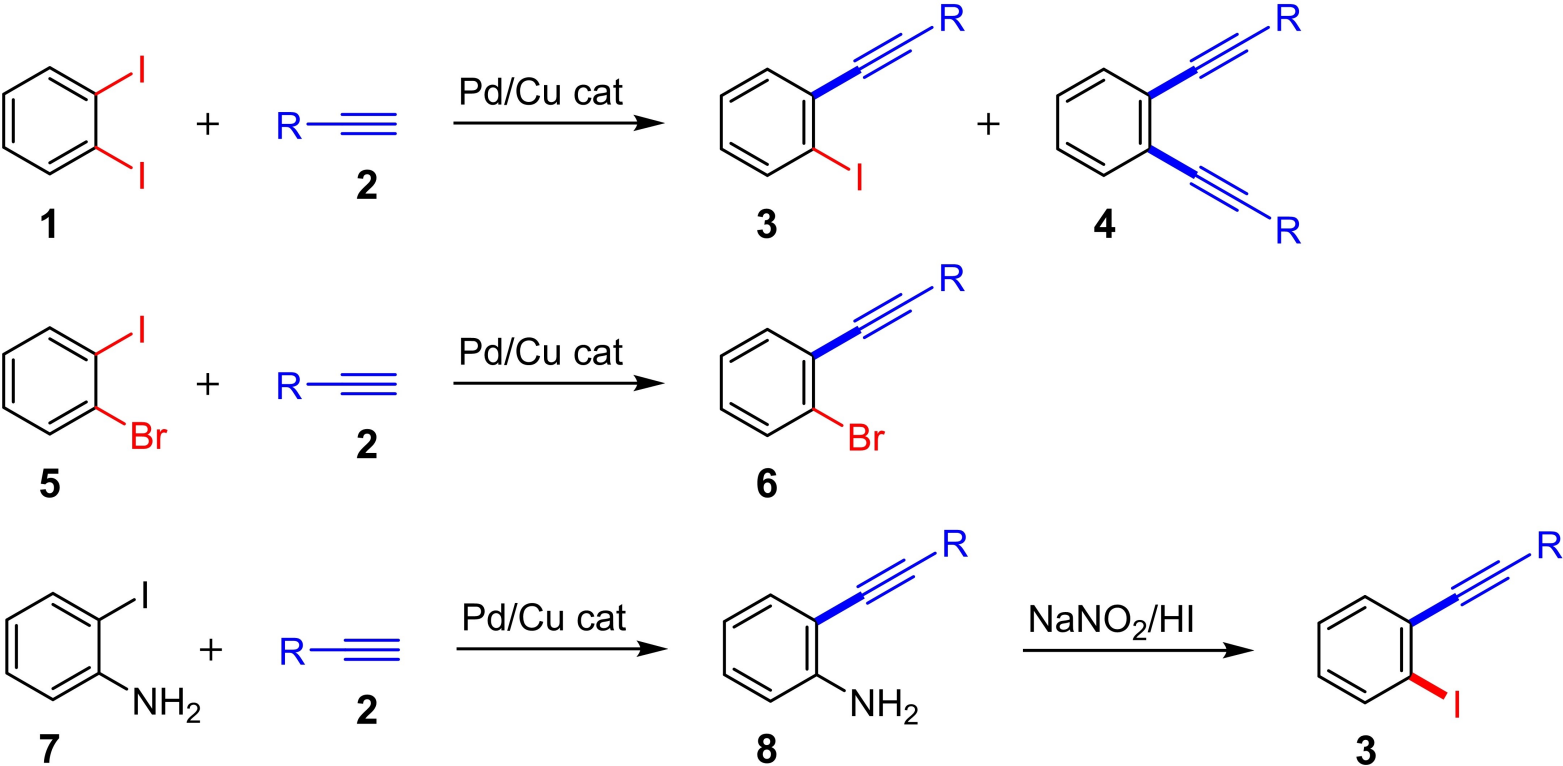
Strategies for the synthesis of *ortho*‐alkynylaryl halides using the Sonogashira reaction.

In synthetic organic chemistry, aryl halides are important building blocks for metal‐catalyzed C−C cross‐coupling reactions.^[6]^ As possible products of these reactions, *ortho*‐alkynylaryl halides (e. g., **3** and **6** in Scheme 2) are important synthons for the rapid and efficient formation of condensed cyclic (hetero)carbon skeletons.[^7^, ^8^] Current synthesis routes for *ortho*‐alkynylaryl halides start from 1,2‐dihaloarene or *ortho*‐iodoaniline derivatives. The desired *ortho*‐alkynylaryl halides can be obtained by a Sonogashira coupling reaction and a subsequent reaction (e. g., a Sandmeyer reaction) (Scheme 2). However, the control of the reaction conditions for the coupling reaction of 1,2‐dihaloarenes for the synthesis of the mono‐alkynylated product is not always trivial. For example, when *ortho*‐diiodobenzene (**1**) is reacted, not only the mono‐alkynylated product **3** but also the bis‐alkynylated product **4** is obtained. One method to overcome this problem is the use of mixed 1,2‐dihaloarenes (e. g., **5**): the more reactive halogen atom is selectively converted in the Sonogashira reaction (Scheme 2). The reaction protocol starting from *ortho*‐iodoaniline (**7**) has two disadvantages: Besides the formation of indoles^[9]^ as by‐products in the Pd/Cu‐catalyzed coupling reaction of **7** to **8**, the harsh reaction conditions of the subsequent Sandmeyer reaction to **3** are often incompatible with sensitive functional groups. Since a multi‐step synthesis also reduces the atom economy, the development of reaction protocols that allow the direct preparation of *ortho*‐alkynylaryl halides is desirable.

**Scheme 3:**
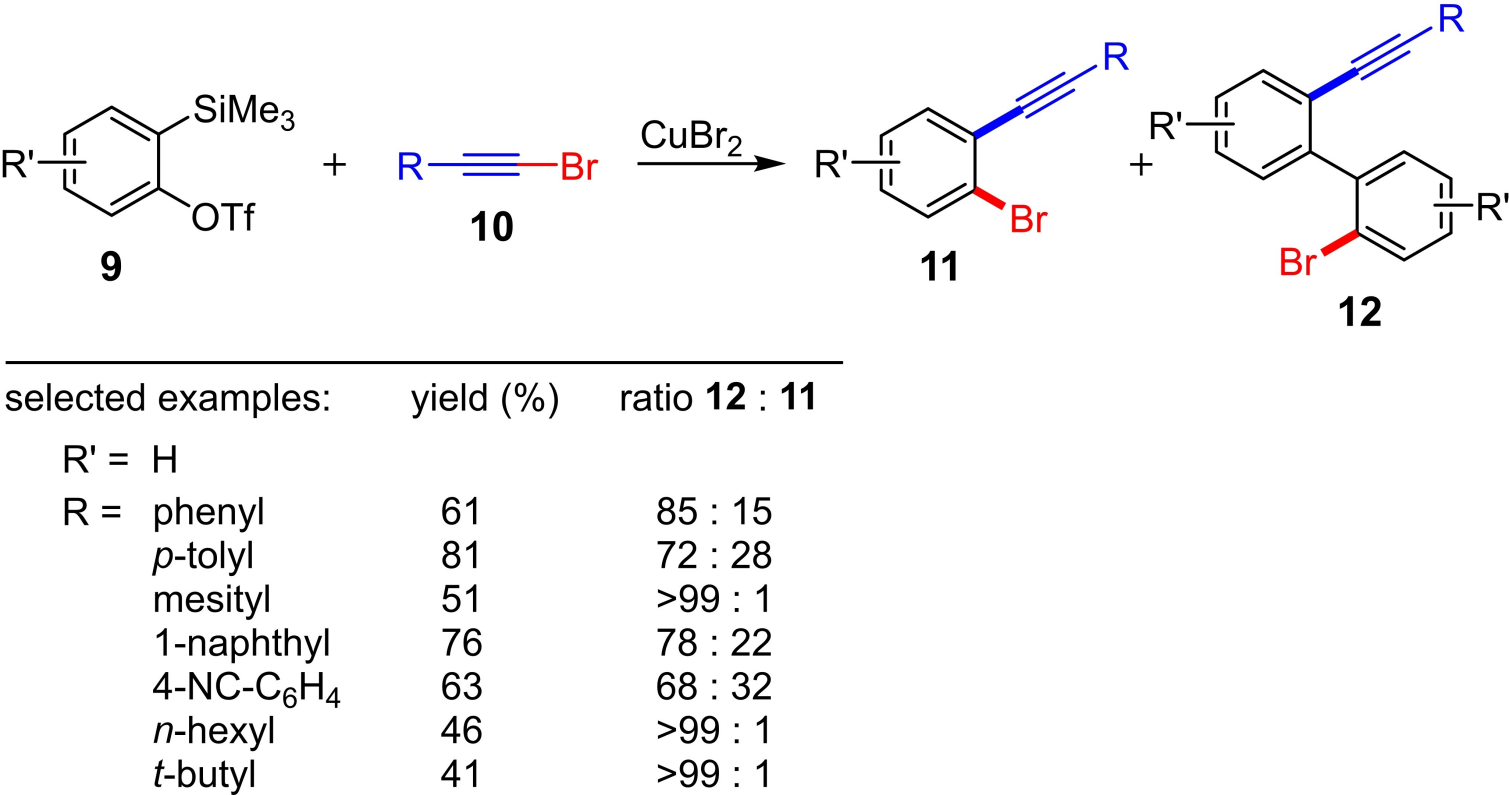
Copper‐catalyzed bromoalkynylation of benzynes. Reaction conditions: CuBr_2_ (20 mol%), KF, 18‐crown‐6, dimethoxyethane (DME), 25 °C.^[1]^

One possibility for the direct synthesis of *ortho*‐alkynylaryl halides is the transition metal‐catalyzed reaction of *ortho*‐silylaryl triflates with bromoalkynes.^[3]^ One example is shown in Scheme 3, which was the first published haloalkynylation reaction of an unsaturated system.^[1]^ Here, the bromoalkynylation of an aryne,^[10]^ which is generated *in situ* from the corresponding *ortho*‐silylaryl triflate using the Kobayashi protocol^[11]^, takes place at room temperature via copper(II) catalysis.^[1]^ From a formal point of view, the aryne inserts into the C(sp)‐Br σ‐bond of the bromoalkyne, which leads to the formation of the two bromoalkynylation products **11** and **12**, whereby **12** being the main product (insertion of two aryne molecules per bromoalkyne) (Scheme 3). The product ratio (max. >99 : 1 of **12 : 11**) and the overall yields of the bromoalkynylation reaction depend on the substituent R of the haloalkyne and on R’ of the *ortho*‐silylaryl triflate; a total of 16 preparative examples with yields of up to 86 % were presented.

**Scheme 4:**
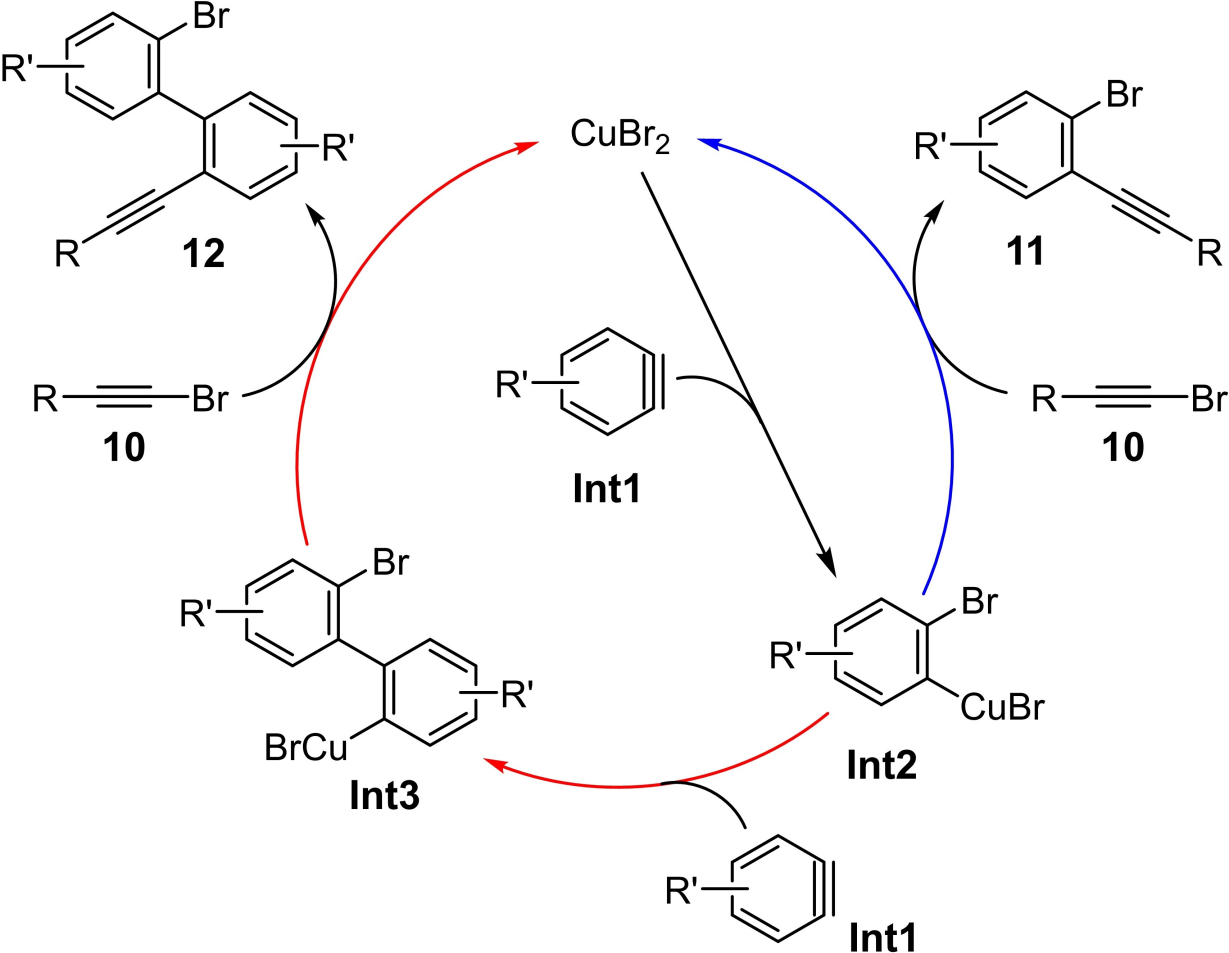
Plausible catalytic cycle for the copper‐catalyzed bromoalkynylation of benzynes **Int1**.^[1]^

Mechanistic investigations indicate that the *in situ* generated aryne **Int1** inserts into a Cu−Br bond of the copper(II) bromide in the first step (Scheme 4); at the same time the second bromide ion is transferred to the *ortho* position yielding the copper(II) aryl species **Int2**. The latter can react in a further step with the haloalkyne **10** to give the 1,2‐bromoalkynylation product **11** (blue arrow in Scheme 4). On the other hand, **Int2** can also react with another aryne molecule **Int1** to form the dimeric bromoalkynylation product **Int3** (red path in Scheme 4). The reaction of this intermediate with the haloalkyne **10** results in the formation of the biphenyl derivative **12**. The authors also investigated the reactivity of the copper(II) aryl species **Int2** regarding the insertion into a C−Br bond of a propargyl and allyl bromide. In both cases the corresponding addition products could be isolated.^[1]^

**Scheme 5:**
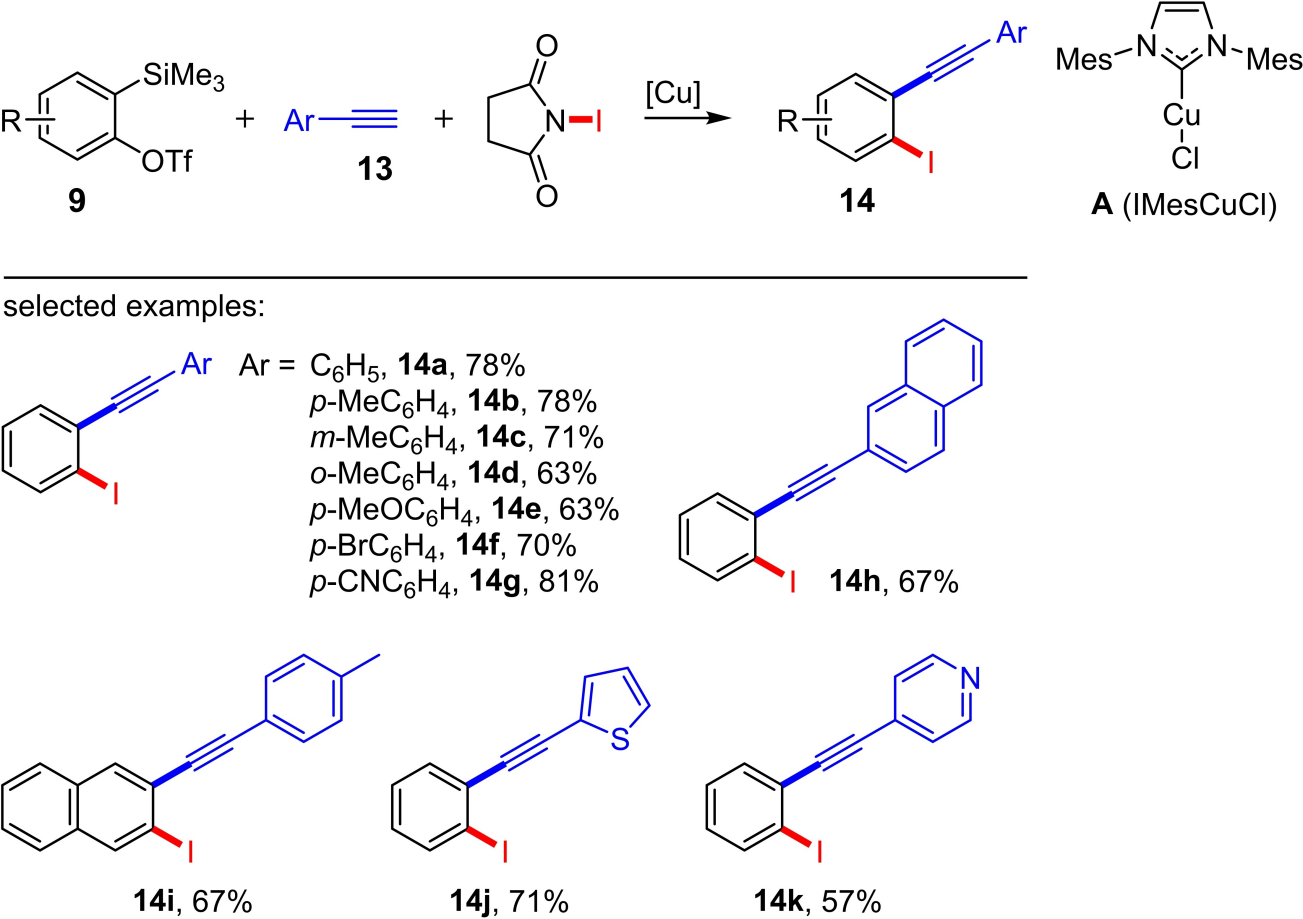
Iodoalkynylation of aromatic compounds using the three‐component reaction of a Kobayashi reagent, an alkyne, and NIS. Reaction conditions: **A** (IMesCuCl) (10 %), Cs_2_CO_3_, CsF, MeCN, 60 °C, 12 h.^[12]^

**Scheme 6:**
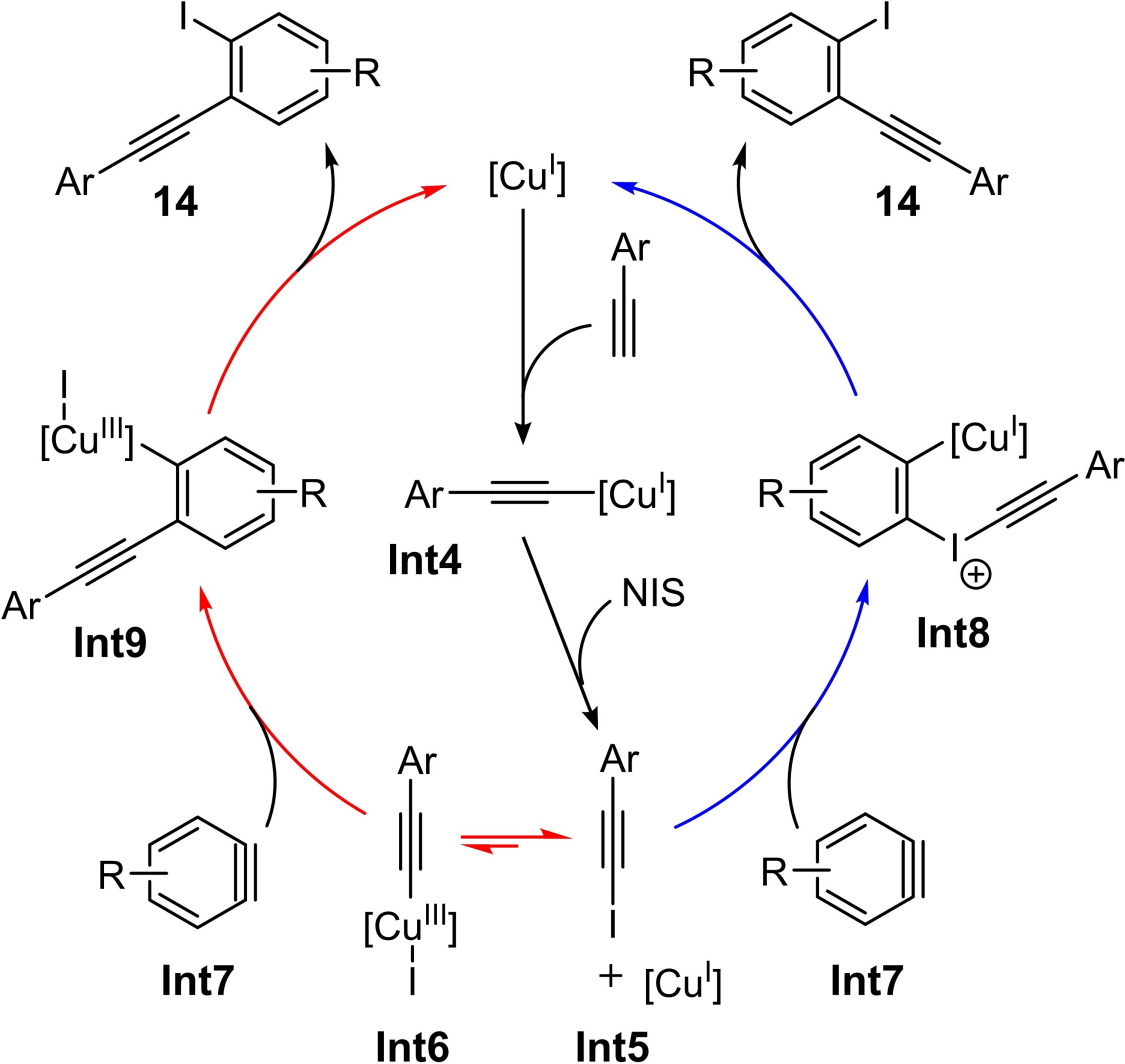
Proposed catalytic cycle for the three‐component reaction of a Kobayashi reagent, an alkyne, and NIS.^[12]^

A recent example is the copper(I)‐catalyzed three‐component 1,2‐iodoalkynylation of arynes (Scheme 5).^[12]^ In this case, the aryne, which is generated *in situ* from the *ortho*‐silylaryl triflate **9**, the terminal alkyne **13** and *N*‐iodosuccinimide (NIS) are converted in the presence of catalytic amounts of a copper(I) complex (10 mol%) at 60 °C in MeCN into the *ortho*‐alkynylaryl iodides **14** (Scheme 5). This reaction protocol enables the selective and (one‐step) synthesis of *ortho*‐alkynylaryl iodides for the first time. Both terminal alkynes with aromatic and heteroaromatic backbone are tolerated. A haloalkynylation with *N*‐bromosuccinimide (NBS) or *N*‐chlorosuccinimide (NCS) has not yet led to the corresponding haloalkynylation product under the optimized reaction conditions.^[12]^ Furthermore, various control experiments were carried out to investigate the underlying reaction mechanism. It could be shown that a base‐promoted (Cs_2_CO_3_) formation of a copper acetylide (**Int4** in Scheme 6) takes place in the first step. This acetylide further reacts in the presence of NIS to form the iodoalkyne **Int5**. Starting from **Int5**, the iodoalkynylation product **14** can be formed via two possible reaction pathways (Scheme 6): Path one (red arrows in Scheme 6) involves the generation of **Int6** via an oxidative addition. A subsequent insertion of aryne **Int7** yields the intermediate **Int9**. The formation of the product **14** subsequently takes place by reductive elimination. Path two (blue arrows in Scheme 6) leads starting from **Int5** to the formation of the copper‐aryliodonium complex **Int8**, from which product **14** is delivered through a 1,3‐alkynyl rearrangement.^[12]^

**Scheme 7:**
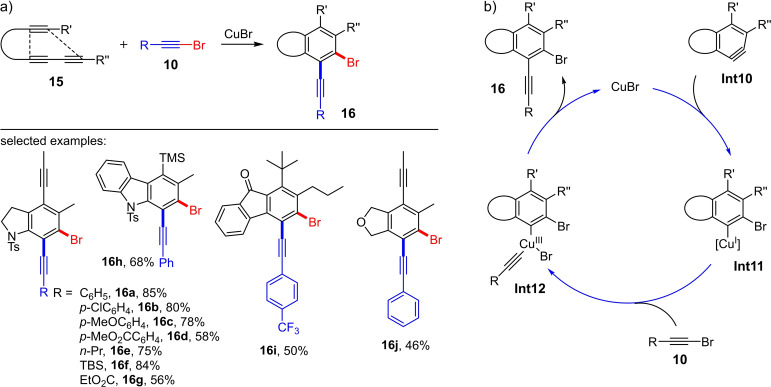
a) Cu(I)‐mediated bromoalkynylation via benzynes generated by the hexadehydro‐Diels‐Alder (HDDA) cycloisomerization reaction. Reaction conditions: CuBr (10 %), MeCN, 80 °C, 16 h. b) Proposed catalytic cycle for the Cu(I)‐catalyzed bromoalkynylation of benzynes **Int9**.^[13]^

Another example for the 1,2‐haloalkynylation of arynes is the copper(I)‐catalyzed reaction (10 mol% CuBr) of the polyynes **15** shown in Scheme 7.^[13]^ The required arynes, which are generated in acetonitrile at 80 °C via a hexadehydro‐Diels‐Alder (HDDA)^[14]^ cycloisomerization, react with the bromoalkynes **10** via copper(I) catalysis to give the corresponding haloalkynylation products **16**. A total of 16 preparative examples with yields of up to 85 % were presented, whereby both the substituents R on the bromoalkynes **10** and the substituents (R’ and R’’) of the polyynes **15** were varied (Scheme 7a). The rate‐limiting step is the thermally induced HDDA cycloisomerization of triyne **15** to aryne **Int10**, which subsequently reacts in a nucleophilic addition with copper(I) bromide to form the copper(I)‐aryl species **Int11** (Scheme 7b). In this step, the halogen atom is transferred to the aromatic unit. The oxidative addition of the bromoalkyne **10** yields the copper(III) complex **Int12**. In the last step, the reductive elimination (elimination of CuBr) takes place with the release of the 1,2‐bromoalkynylation product **16** (Scheme 7b). The mechanism is supported by the fact that when using copper(I) chloride or iodide instead of copper(I) bromide, traces of the corresponding chloro‐ or iodoalkynylation product could be observed. Thus, the bromine atom in **16** stems from the catalytic species. A glance at the proposed catalytic cycle shows that a bromine anion is added to aryne **Int11** in the first step, which determines the regioselectivity. When describing the reaction using synthons (see Scheme 1), this would correspond to the addition of a halide and an alkynyl cation (synthon III).

## 3 Haloalkynylation Reactions of Alkenes (Direct Synthesis of Homopropargyl Halides)

**Scheme 8:**
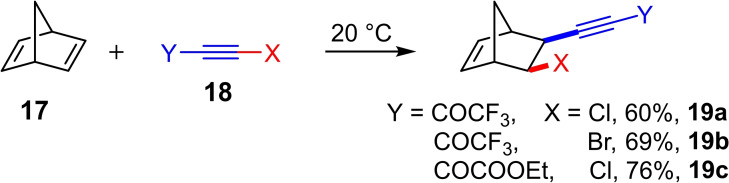
Haloalkynylation of norbornadiene (**17**) with the haloacetylenes **18**.^[15]^

The haloalkynylation of alkenes was first reported in 2009 by Koldobskii et al.^[15]^ They observed a 1,2‐haloalkynylation of norbornadiene (**17**) at room temperature using the chloro‐ and bromoalkynes **18**, which are activated by either a trifluoroacetyl or ethyloxalyl group (Scheme 8). The corresponding products of the 1,2‐haloalkynylation, the homopropargyl halides **19** are versatile compounds. Although no mechanistic investigations were carried out, the authors postulated a cyclic halonium ion as intermediate, which later turned out to be an important intermediate in gold(I)‐catalyzed 1,2‐haloalkynylations of alkenes.

**Scheme 9:**
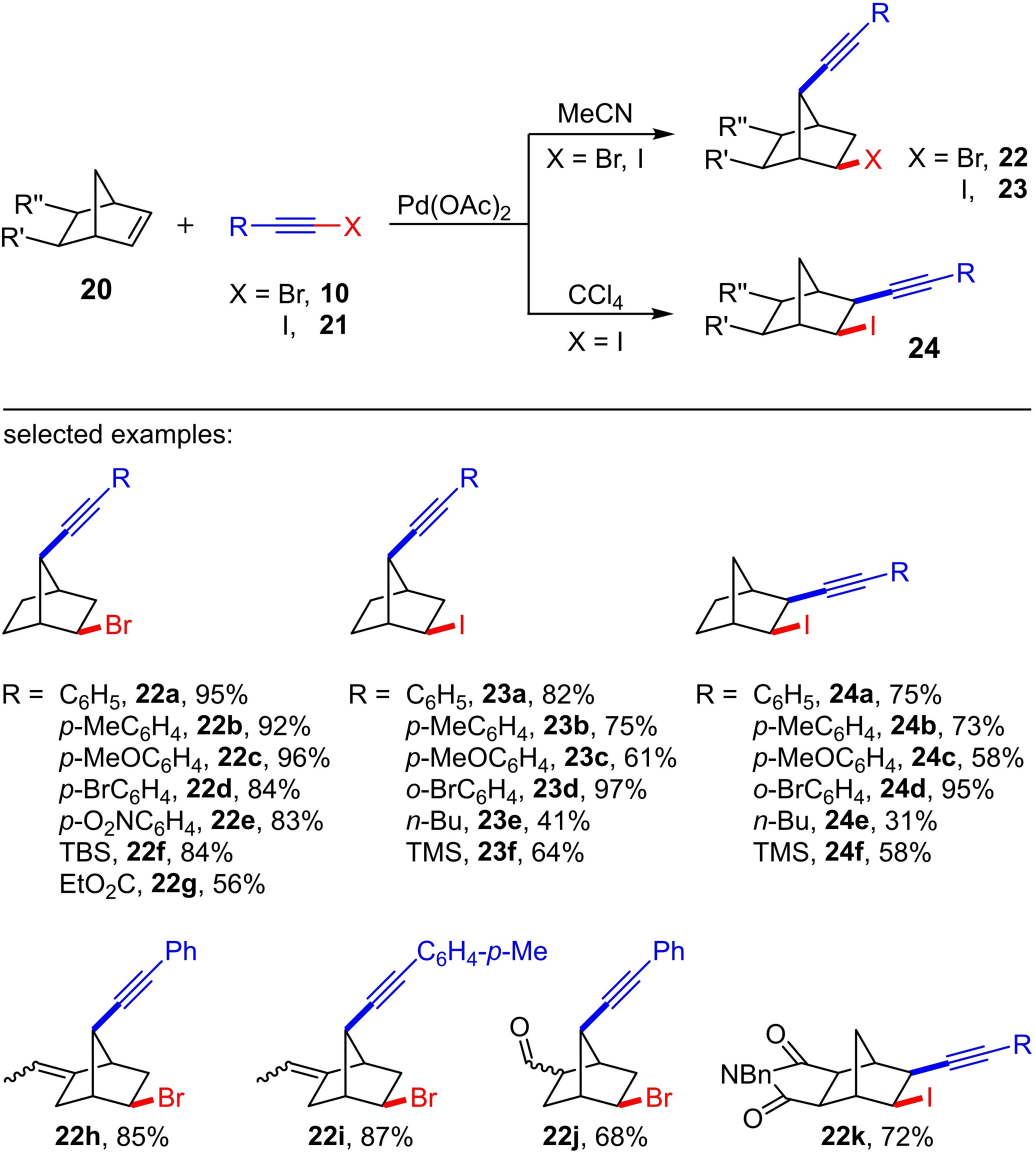
Pd‐catalyzed haloalkynylation of the norbornene scaffolds **20** depending on the solvent. Reaction conditions: 50 °C, 12 h, Pd(OAc)_2_ (5‐10 mol%).[^16^, ^17^]

Two years later, the reactivity of C−C double bonds towards haloalkynes was investigated more intensively by Jiang et al. via palladium catalysis.^[16]^ During the reaction of the norbornene derivatives **20** with the bromoacetylenes **10** using Pd(OAc)_2_ as catalyst in acetonitrile at 35 °C, they were able to observe the formation of the bromoalkynylation products **22** (Scheme 9). In contrast to the investigations by Koldobskii et al., the homopropargyl bromides **22** represent the C7‐functionalized products. That means a 1,7‐haloalkynylation reaction takes place. A total of 26 examples with preparative yields of up to 95 % were presented. Further investigations with cyclooctene instead of norbornene as starting material led exclusively to the [2+2] cycloaddition product. This demonstrates that the norbornene system must play a crucial role in the mechanism. It was therefore assumed that the reaction occurs via a non‐classical carbocation (norbornonium cation) and the C‐7 functionalization can be achieved through a nucleophile rearrangement.

Later, Tong et al. investigated the Pd‐catalyzed haloalkynylation of norbornene in more detail using the iodoalkynes **21** as haloalkynes.^[17]^ The reaction of iodophenylacetylene and norbornene with 10 mol% Pd(OAc)_2_ as catalyst at 50 °C yielded depending on the solvent either the 1,7‐iodoalkynylation product **23 a** (in acetonitrile) or the 1,2‐iodoalkynylation product **24 a** (in tetrachloromethane) as main product (Scheme 9). In both cases, a product mixture is formed, whereby the ratio depends on the nature of the substituent R of the iodoalkyne **21**: The product ratio for **23 : 24** of the Pd‐catalyzed haloalkynylation of norbornene ranges in CCl_4_ from 99 : 1 to 81 : 19 (12 examples; preparative yields for **23** up to 97 %). The analogous reactions in MeCN lead to a product ratio of 99 : 1 to 80 : 20 for **24 : 23** (preparative yields for **24** amounts up to 95 %). The conversion of phenylbromoacetylene and norbornene led to the 1,7‐bromoalkynylation product **22 a** under the optimized reaction conditions (see Jiang et al.^[16]^). In contrast to the reaction with phenyliodoacetylene, no reaction to the corresponding 1,2‐bromoalkynylation product could be observed in CCl_4_.

Interestingly, Tong et al. converted a norbornene derivative showing a succinimide backbone with phenyliodoacetylene under the optimized reaction conditions in MeCN and obtained almost exclusively the 1,2‐iodoalkynylation product **22 k** (Scheme 9; 72 % yield; 97 % selectivity). This suggests that a common intermediate occurs in the 1,2‐ and 1,7‐iodoalkynylation, however, in this case (**22 k**), the decisive rearrangement process for the 1,7‐iodoalkynylation is probably prevented due to the rigid molecular structure of the norbornene derivative.^[17]^ To gain insight into the reaction mechanism, Tong et al. carried out the Pd(OAc)_2_‐catalyzed reaction of iodophenylacetylene and norbornene in MeCN in the presence of 5 equivalents of LiBr. In addition to the 1,7‐iodoalkynylation product (**23 a**, 40 %) and the 1,2‐iodoalkynylation product (**24 a**, 9 %), the corresponding 1,7‐bromoalkynylation product (51 %) was also formed. Hence, it can be assumed that the halogen exchange occurs prior to the formation of the 1,7‐haloalkynylation product. In contrast, the formation of the corresponding 1,2‐bromoalkynylated product could not be detected in the presence of LiBr, i. e. no halogen exchange is likely involved in the 1,2‐haloalkynylation. Furthermore, the authors assume that a Pd(0) species occurs at the beginning of the catalytic cycle in the Pd‐catalyzed iodoalkynylation of norbornene, since the reaction also works with classical Pd(0) systems such as Pd(PPh_3_)_4_.^[17]^

**Scheme 10:**
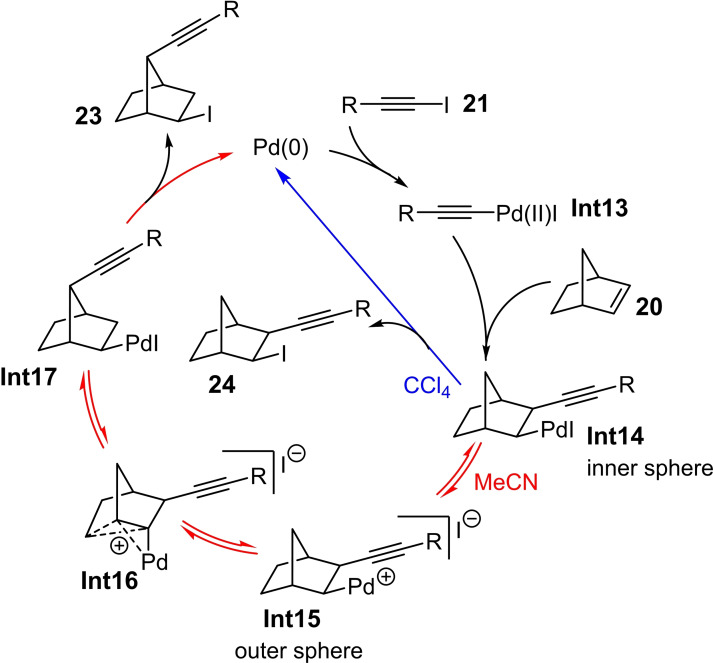
Proposed catalytic cycle for the Pd‐catalyzed haloalkynylation of norbornene scaffolds **20** depending on the solvent.^[17]^

Based on the experimental data mentioned above, the authors describe the catalysis cycle as follows: In the first step, the oxidative addition of the Pd(0) species to the iodoalkyne **21** leads to the formation of the Pd(II)acetylide **Int13** (Scheme 10). The alkene **20** subsequently coordinates to the Pd(II) center, which follows a *cis*‐insertion into the Pd−C bond of the Pd(II) alkyne complex **Int13** yielding **Int14**. The influence of the solvent on the outcome of the reaction (i. e., 1,2‐ vs. 1,7‐iodoalkynylation product) can be illustrated using the ion pairs **Int14** (inner sphere) and **Int15** (outer sphere), which are at equilibrium (Scheme 10). In the presented mechanism, the palladium center is considered neutral for the inner form, whereas the outer form **Int15** represents a cationic palladium species. Thus, in nonpolar solvents such as CCl_4_, the form **Int14** dominates, which preferentially gives the 1,2‐iodoalkynylation product **24** by reductive elimination (blue route in Scheme 10). Unlike in polar media such as MeCN, the cationic species **Int15** can be better stabilized. As a result, the norbornene skeleton is more positively charged, so that the nonclassical carbocation **Int16** is generated. The latter is converted into the neutral Pd complex **Int17** via a skeletal rearrangement and re‐coordination of the iodide ligand (red path in Scheme 10). Starting from **Int17**, the 1,7‐iodoalkynylation product **23** is formed by reductive elimination.

**Scheme 11:**
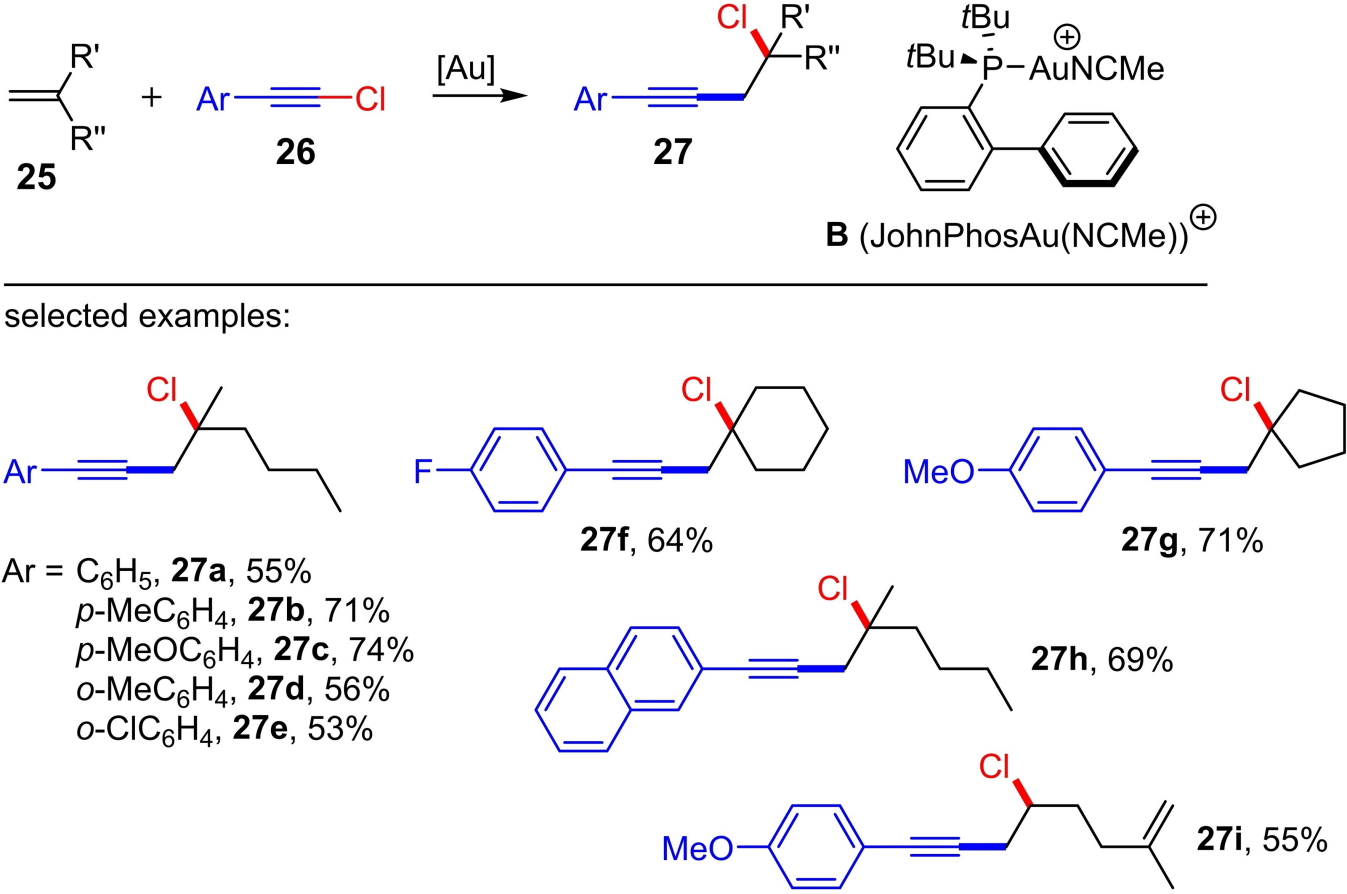
Gold(I)‐catalyzed chloroalkynylation of 1,1‐disubstituted alkenes with chloroarylacetylenes. Reaction conditions: **B** ⋅ SbF_6_^−^ ([JohnPhosAu(NCMe)]SbF_6_) (5 mol%), DCM, room temperature.^[18]^

Until recently, all thermal and metal‐catalyzed haloalkynylation reactions of alkenes were limited to norbornene and norbornene derivatives. In 2019 Kreuzahler and Haberhauer reported for the first time a haloalkynylation of substituted alkenes using gold(I) as catalyst.^[18]^ In this study, the 1,1‐disubstituted alkenes **25** and the aryl‐substituted chloroacetylenes **26** are converted into the corresponding homopropargyl chlorides **27** in dichloromethane (DCM) at room temperature using the cationic gold(I) phosphine complex [JohnPhosAu(NCMe)]SbF_6_ (5 mol%) (Scheme 11). A total of 14 preparative examples with yields of up to 74 % were presented.

**Scheme 12:**
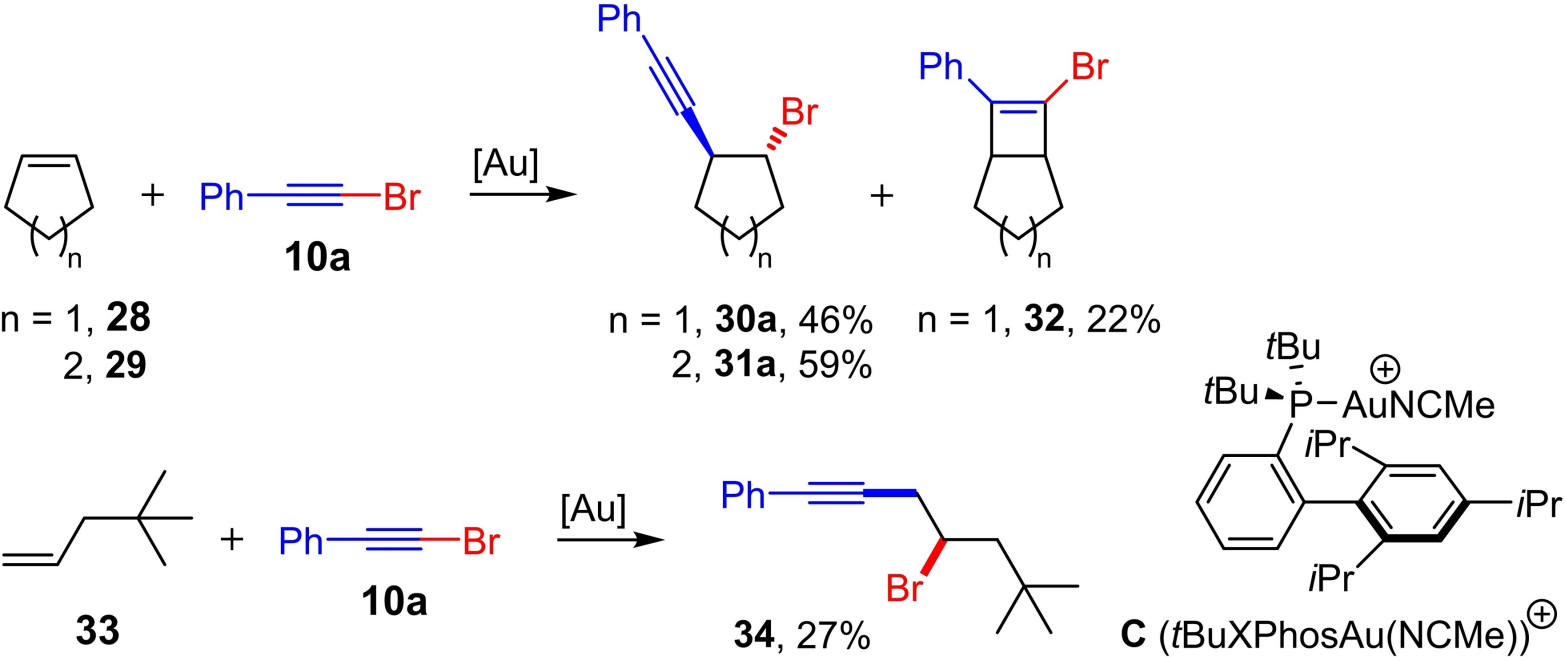
Gold(I)‐catalyzed bromoalkynylation of alkenes with bromophenylacetylene (**10 a**). Reaction conditions: **B** ⋅ BArF_24_^−^ ([*t*BuXPhosAu(NCMe)]BArF_24_) (3 mol%), DCM (1 m), 23 °C.^[19]^

As part of the work on the gold(I)‐catalyzed synthesis of 1,4‐enynes by the reaction of allylsilanes and aryl‐substituted bromoalkynes, Echavarren et al. also tested mono‐substituted (**33**) and cyclic 1,2‐disubstituted alkenes (**28** and **29**) (Scheme 12).^[19]^ They were able to show that the bromoalkynylation of alkene **33** leads regioselectively to the homopropargyl bromide **34**. Furthermore, the addition of **10 a** to cyclopentene (**28**) or cyclohexene (**29**) proceeds stereoselectively to the *anti*‐bromoalkynylation products **30 a** and **31 a**, respectively. Interestingly, when cyclopentene (**28**) is used, the [2+2] cycloaddition product **32** is also formed besides the bromoalkynylation product **30 a**. This does not occur with cyclohexene as a reactant.

**Scheme 13:**
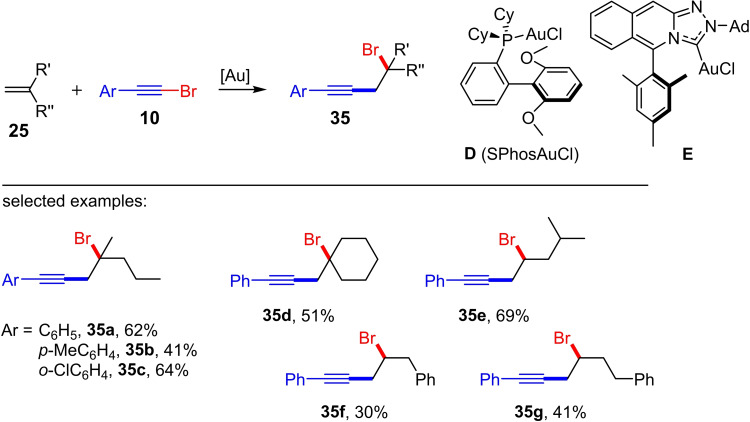
a) Gold(I)‐catalyzed bromoalkynylation of alkenes with bromoarylacetylenes. Reaction conditions: **D** (SPhosAuCl) (5 mol%), NaBArF_24_ (10 mol%) or **E** (2.5 mol%), NaBArF_24_ (2.5 mol%), CHCl_3_, room temperature.^[20]^

Only a few months later, the gold(I)‐catalyzed haloalkynylation of cyclic and acyclic terminal alkenes was also investigated by Fernández, Lassaletta et al.^[20]^ They were able to show that by varying the catalyst system (ligand and counterion) and using the aryl‐substituted bromoalkynes **10**, both 1,1‐disubstituted (R’ and R’’≠H) and mono‐substituted alkenes (R’=H) can be converted to the corresponding homopropargyl bromides **35** in CHCl_3_ as solvent (Scheme 13).^[20]^ For this purpose, the authors used two different catalyst systems based either on a carbene ligand (see **E** in Scheme 13) or on a phosphine ligand (SPhos); the BArF_24_^−^ anion (2.5–10 mol% NaBArF_24_) is used as the counterion in both cases. The **E**/NaBArF_24_ system was used for the gold(I)‐catalyzed bromoalkynylation of 1,1‐disubstituted alkenes. A total of six reaction examples with preparative yields of up to 64 % were presented (Scheme 13). In contrast, the use of the SPhosAuCl/NaBArF_24_ system only allows the bromoalkynylation of mono‐substituted alkenes (R’=H, four examples with yields between 30–69 %). An extension of these reaction conditions to the corresponding aryl‐substituted chloroalkynes showed that here again only the 1,1‐disubstituted (R’ and R’’≠H) alkenes can be converted to the homoprogargyl chlorides. For example, the gold(I)‐catalyzed reaction of chlorophenylacetylene with 4‐methyl‐1‐pentene leads exclusively to the [2+2] cycloaddition product.^[20]^ This is in line with the results by Kreuzahler and Haberhauer, who were only able to observe a 1,2‐chloroalkynylation for the 1,1‐disubstituted systems.^[18]^

**Scheme 14:**
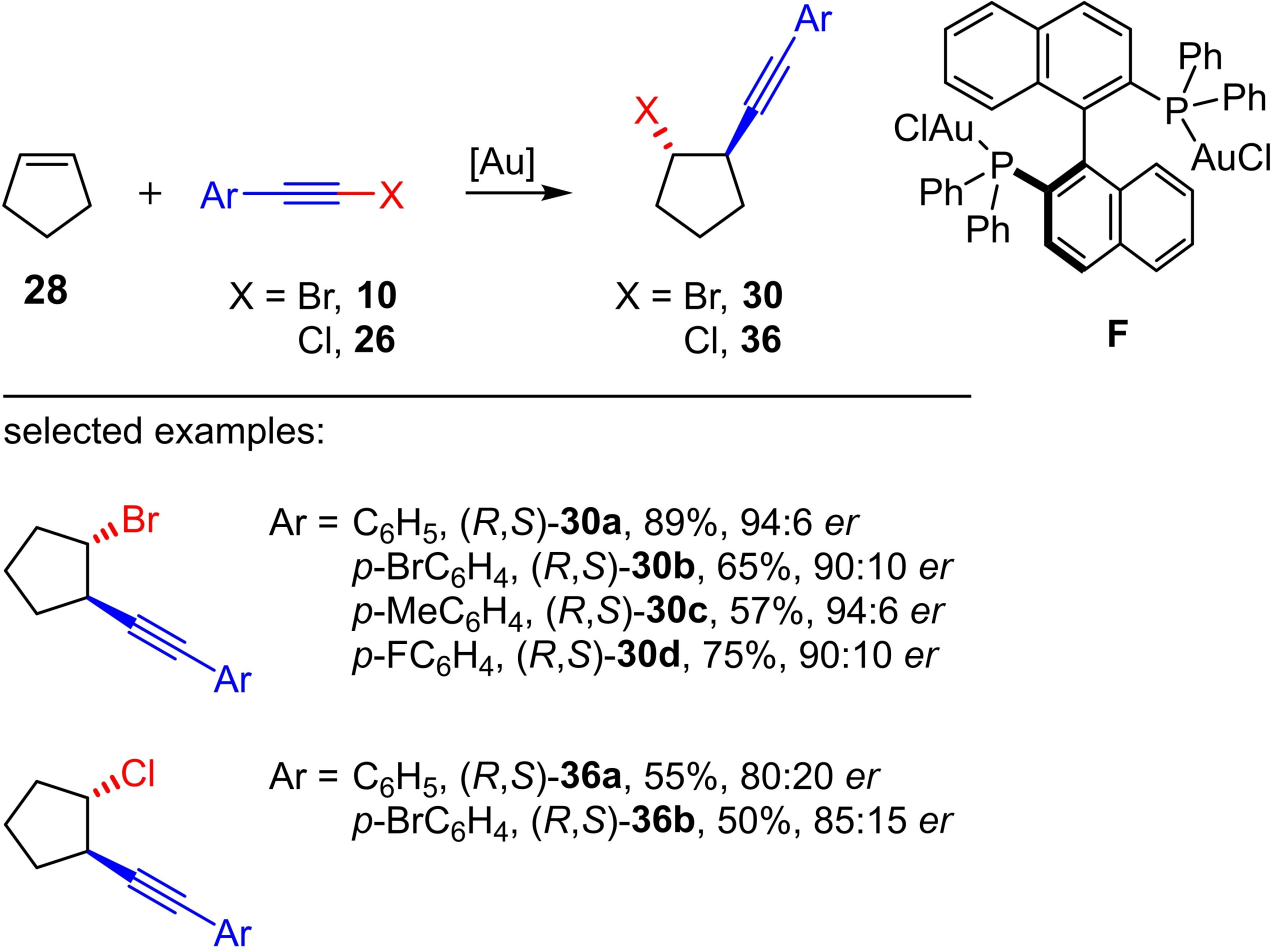
Enantioselective gold(I)‐catalyzed haloalkynylation of cyclopentene with haloarylacetylenes. Reaction conditions: (*S*)‐**F** (2.5 mol%), NaBArF_24_ (2.5 mol%), CHCl_3_.^[20]^

In principle, the haloalkynylation of alkenes leads to homopropargylic halides showing one or two new stereogenic centers. The starting point for the asymmetric gold(I)‐catalyzed haloalkynylation of cyclic alkenes was the reaction of phenylbromoacetylene (**10 a**) and cyclopentene (**28**) (Scheme 14).^[20]^ While the gold(I)‐catalyzed reaction of phenylbromoacetylene (**10 a**) with cyclopentene (**28**) leads to the *anti*‐product **30 a** with a preparative yield of 90 %, the reaction of phenylbromoacetylene (**10 a**) with cyclooctene delivered exclusively the [2+2] cycloaddition product. The *anti*‐orientation of the substituents in **30 a** was elucidated by means of ^1^H NMR spectroscopy^[19]^ and crystal structure analysis.^[20]^

The reaction of cyclopentene (**28**) with the phenyl‐substituted chloroacetylene **26 a** gave the corresponding haloalkynylation product **36 a** under the optimized reaction conditions, albeit in a lower yield (55 %) compared to the bromoalkyne **30 a** (Scheme 14). The reaction of cyclohexene and cyclooctene with the phenyl‐substituted chloroacetylene **26 a** only led to the corresponding [2+2] cycloaddition products. In other words, both the yield and the selectivity (alkynylation product vs. cycloaddition product) of the gold(I)‐catalyzed haloalkynylation of cyclic alkenes are decisively determined by the ring size of the alkene, the polarization of the C−X bond of the haloalkyne (X=Cl vs. X=Br) as well as the electronic nature of the ligand. Furthermore, Fernández, Lassaletta et al. could impressively show that the gold(I)‐catalyzed bromoalkynylation of cyclopentene can also be performed enantioselectively (a maximum *er* of 94 : 6) by using the chiral phosphine ligand **F** (Scheme 14). The selectivity (*er* in %) was determined by means of chiral HPLC. The corresponding enantioselective chloroalkynylation of cyclopentene, however, gave significantly low *er* values (max. 85 : 15). The product of the enantioselective bromoalkynylation was subsequently reacted with NaN_3_ as nucleophile in a substitution reaction with inversion of the configuration, whereby no racemization was observed. The conversion of the azide with an acetylene afforded a triazole via click reaction. From the latter crystals suitable for single‐crystal X‐ray diffraction analysis were obtained, which were used to assign the absolute configuration of the triazole and hence of the homopropargylic bromide.^[20]^

**Scheme 15:**
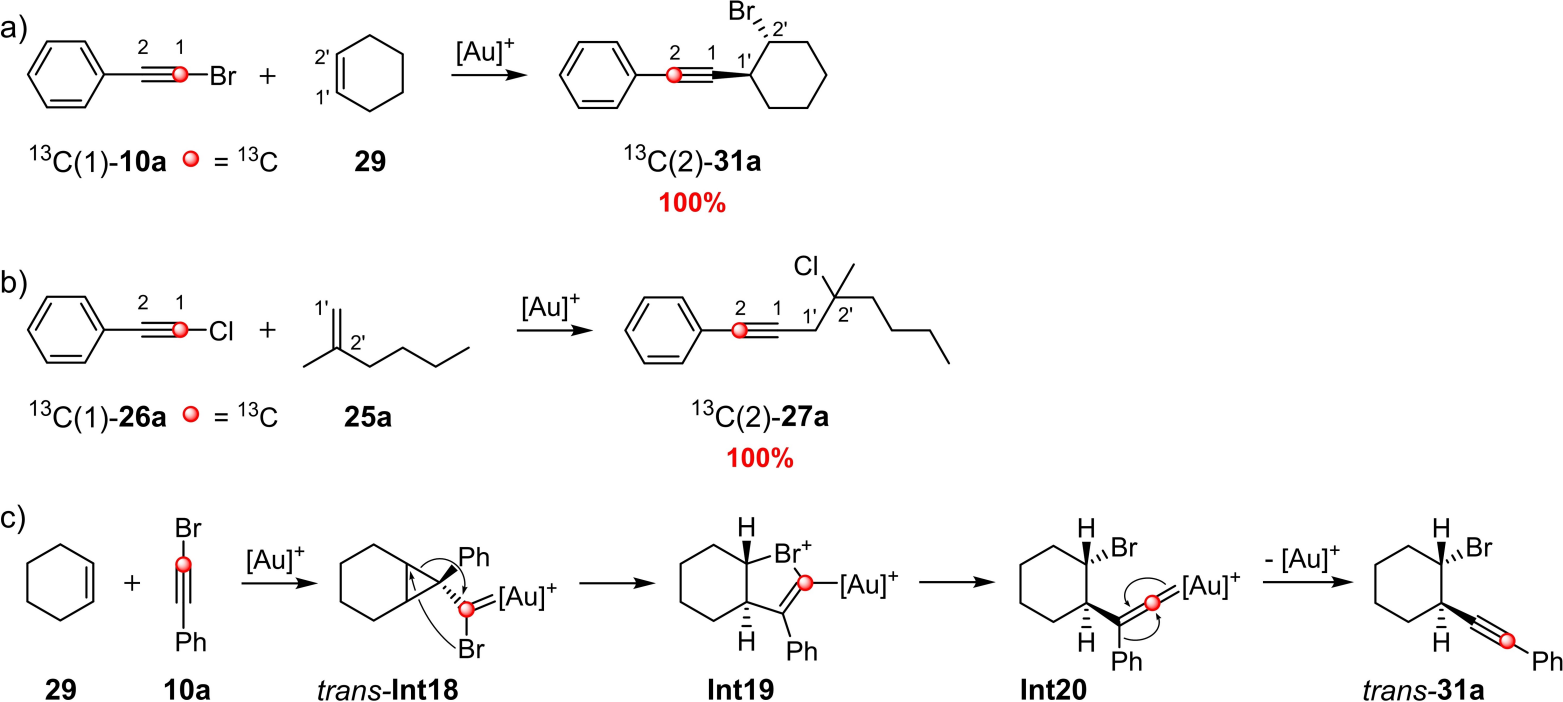
Investigation of the reaction mechanism of the gold(I)‐catalyzed 1,2‐haloalkynylation of alkenes with ^13^C‐labeled starting materials. As ligands for the gold(I) complexes, *t‐*BuXPhos and JohnPhos were used.[^18^, ^19^]

To get an insight into the reaction mechanism, Echavarren et al. investigated the gold(I)‐catalyzed reaction of ^13^C‐labeled phenylbromoacetylene (^13^C‐**10 a**) and cyclohexene (**29**) (Scheme 15a).^[19]^ Analysis of the bromoalkynylation product **31 a** by means of ^13^C NMR spectroscopy showed that a rearrangement must have taken place, as the ^13^C‐labeled acetylenic carbon atom had changed position. On the basis of their investigations on the gold(I)‐catalyzed reaction of allylsilanes and bromoalkynes, a plausible mechanism was proposed that explained the preferred formation of the *anti*‐product **31 a** by taking the ^13^C labeling experiment into account (Scheme 15c):^[19]^ In the first step, the cyclopropylgold(I) carbene species **Int18** is generated, which converts into the *trans*‐bicyclic bromonium ion **Int19** via an S_N_2‐like reaction accompanied by inversion of the configuration. The opening of the bromonium ion and rearrangement of the phenyl group, which explains the change in position of the ^13^C‐labeled carbon atom, leads to the formation of the product *trans*‐**31 a**.

Kreuzahler and Haberhauer studied the gold(I)‐catalyzed chloroalkynylation of 1,1‐disubstituted alkenes in more detail using DFT calculations and ^13^C labeling experiments.^[18]^ They, too, were able to observe a change in position of the ^13^C‐labeled carbon atom during the gold(I)‐catalyzed reaction of the ^13^C‐labeled chloroalkyne **26 a** with the alkene **25 a** (Scheme 15b). The ^13^C‐labeled chloroalkynylation product **27 a** was isolated with a yield of 64 %; the proportion of the ^13^C‐labeled product at position C2 was 100 %.

**Scheme 16:**
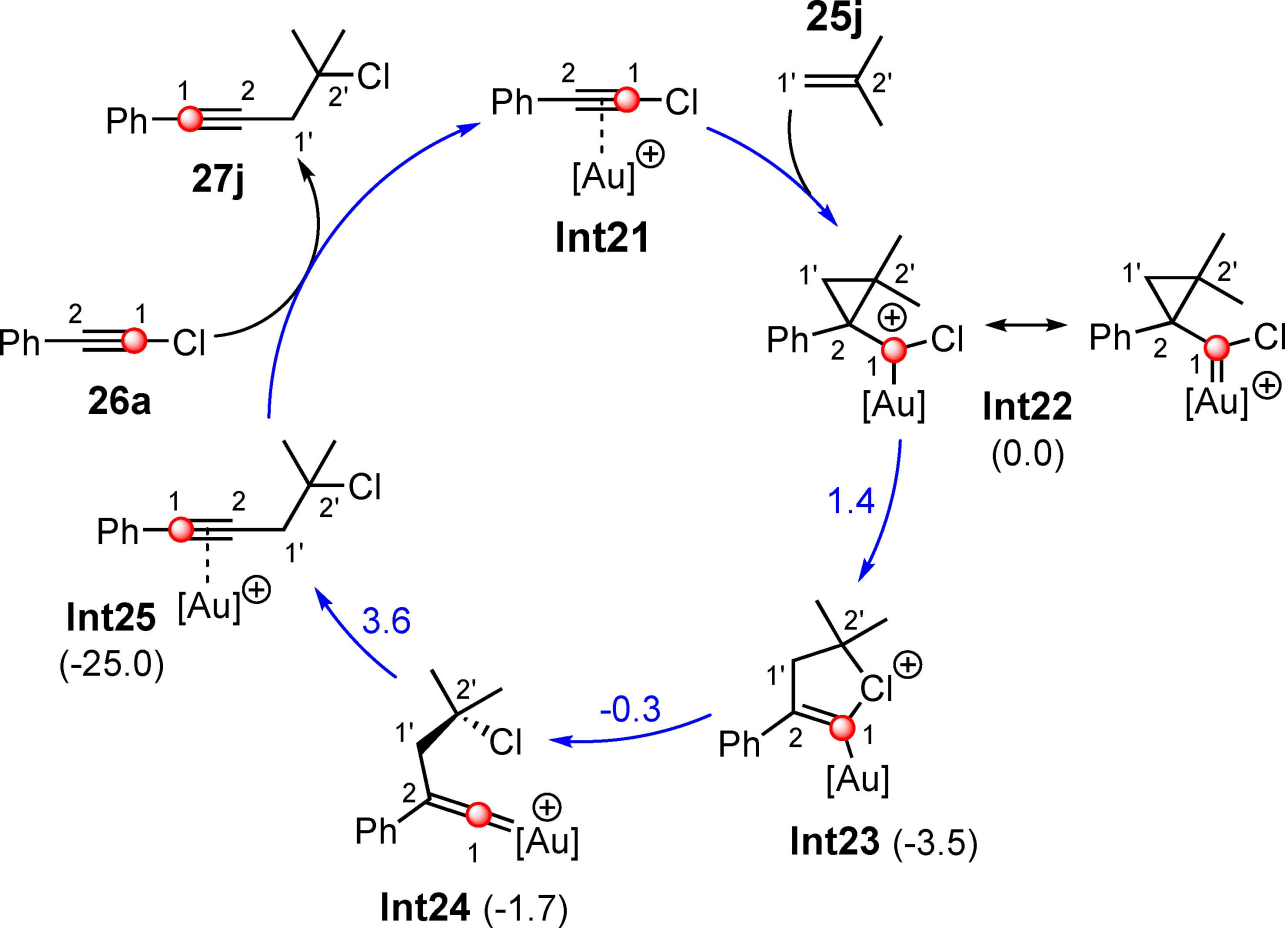
By means of B3LYP‐D3BJ(SMD) calculated catalytic cycle for the gold(I)‐catalyzed 1,2‐chloroalkynylation of isobutene (**25 j**) with chlorophenylacetylene (**26 a**). The free energies (Δ*G*) of the intermediates **Int23‐Int25** (black numbers) and transition states (blue numbers) are given in kcal/mol and are relative to **Int22**.^[18]^ [Au]^+^=JohnPhosAu^+^.

For the quantum chemical calculations, the reaction of chlorophenylacetylene (**26 a**) and isobutene (**25 j**) in the presence of the cationic JohnPhosAu complex was chosen (Scheme 16).^[18]^ In principle, alkene **25** can attack the cationic gold(I) alkyne complex **Int21** on both the C1 carbon atom and the C2 carbon atom. An attack on the C1 atom of **Int21** leads to a stable intermediate, but the quantum chemical calculations suggest that the attack on the C2 atom of **Int21** is preferred by more than 6 kcal/mol. The reason for this is that the positive charge in the transition state can be stabilized by the adjacent phenyl ring. Attacking the C2 atom of **Int21** leads to cyclopropyl gold(I) carbene **Int22**, from which several reaction pathways are possible. Quantum chemical calculations revealed that one of the three possible routes is greatly preferred in terms of energy. Therefore, only the energetically preferred one will be discussed in the following (Scheme 16). In the first step of this reaction path, the three‐membered ring of **Int22** undergoes a ring‐opening and the cyclic halonium ion **Int23** is generated. The activation barrier is very low at 1.4 kcal/mol. A renewed ring‐opening yields the gold(I) vinylidene species^[21]^ **Int24** in the following step. The phenyl substituent subsequently rearranges to form the alkyne gold(I) complex **Int25**. This rearrangement of the phenyl ring requires only a low activation barrier (5.3 kcal/mol). The transfer of the catalytic species to a new haloalkyne molecule **26 a** yields the homopropargyl halide **27 j** and closes the catalytic cycle (Scheme 16). These results are in agreement with the ^13^C labeling experiment, as 100 % rearranged product was found in this experiment. If one considers the regioselectivity for the Au(I)‐catalyzed haloalkynylation of alkenes presented above, one recognizes that this can best be described as the addition of an alkynyl cation and a halogen anion (synthon III see Scheme 1): The cation attacks the less substituted carbon atom, which is why the addition of the chloride takes place on the more substituted carbon atom.

**Scheme 17:**
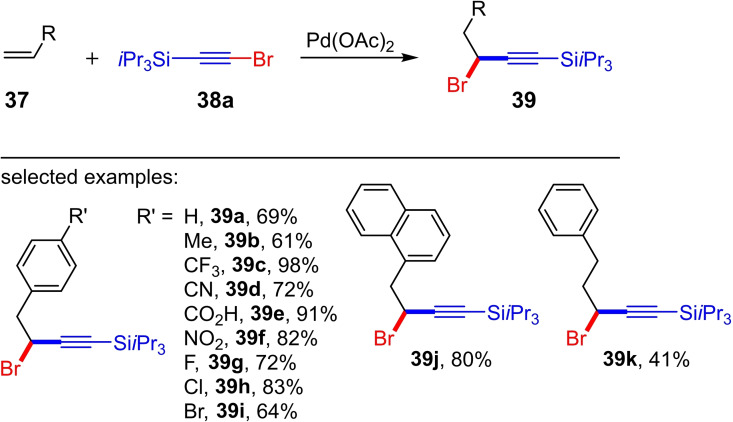
Palladium‐catalyzed 1,1‐bromoalkynylation of terminal alkenes with the silyl‐protected alkynyl bromide **38 a**. Reaction conditions: toluene, 75 °C, 20 h, Pd(OAc)_2_ (10 mol%).^[22]^

Very recently, Ano et al. reported the first Pd‐catalyzed haloalkynylation of terminal alkenes (Scheme 17).^[22]^ Surprisingly, in contrast to the Pd‐catalyzed 1,2‐haloalkynylation of norbornene derivates[^16^, ^17^] and the gold‐catalyzed 1,2‐haloalkynylation of terminal alkenes,[^18^, ^19^, ^20^] their protocol delivers exclusively the 1,1‐haloalkynylation product **39** (Scheme 17). The reaction of the TIPS‐substituted bromoalkyne **38 a** and the terminal alkene **37** is conducted in the presence of 10 mol% Pd(OAc)_2_ in toluene at 75 °C and delivers the 1,1‐bromoalkynylation product **39** (>30 examples; preparative yields for **39** up to 98 %). Mechanistical DFT studies and deuterium‐labelling experiments indicate a Pd(0)/Pd(II) catalytic cycle.^[22]^

In summary, the 1,2‐haloalkynylation of norbornene and norbornene derivatives with aryl‐ and alkyl‐substituted iodo‐ and bromoalkynes as well as the 1,1‐bromoalkynylation of terminal alkenes is currently possible using palladium catalysis. The use of cationic gold(I) complexes enabled the bromo‐ and chloroalkynylation of cyclic 1,2‐disubstituted (cyclopentene, cyclohexene) as well as acyclic 1,1‐disubstituted and mono‐substituted alkenes for the first time. Tri‐ and tetra‐substituted alkenes could not be converted to the corresponding haloalkynylation products with either palladium or gold catalysis. A disadvantage of the gold(I)‐catalyzed variant is that the haloalkynylation reaction has so far been limited to aryl‐substituted haloalkynes.

## 4 Haloalkynylation Reactions of Alkynes (Direct Synthesis of Halogenated Enynes)

The formation of C(sp)−C(sp^2^) bonds is an important strategy for the synthesis of differently conjugated systems and biologically active compounds.^[6]^ Especially higher‐substituted conjugated enynes with functional groups are of great interest as versatile synthesis intermediates. In general, the synthesis takes place in several steps and includes coupling reactions of substituted alkenes and terminal alkynes.^[23]^ In addition, side reactions with the catalyst system can occur with sensitive functional groups, which increases the synthetic effort.

**Scheme 18:**
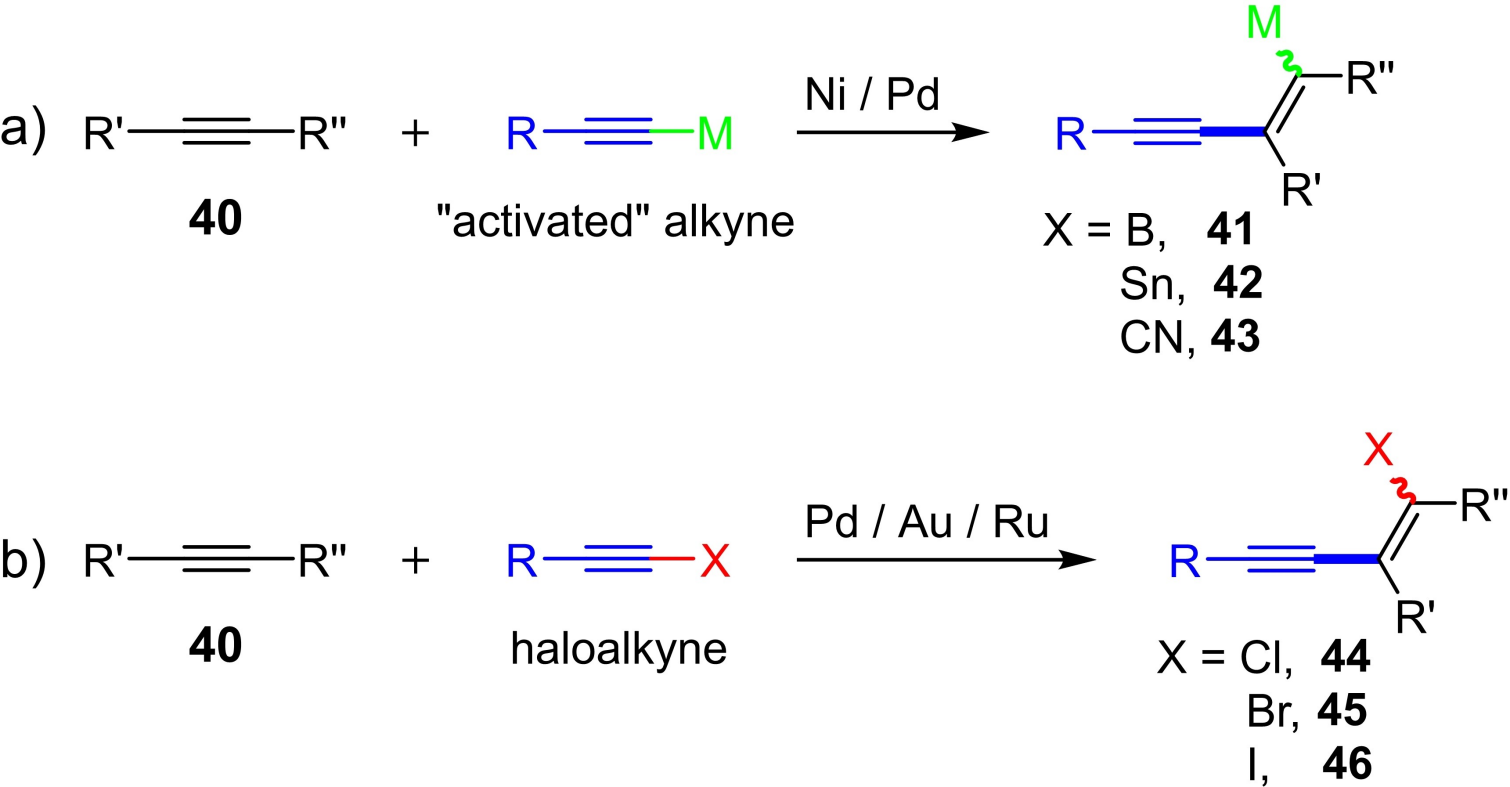
Synthesis of functionalized enynes starting from activated alkynes (a) and haloalkynes (b).

**Scheme 19:**
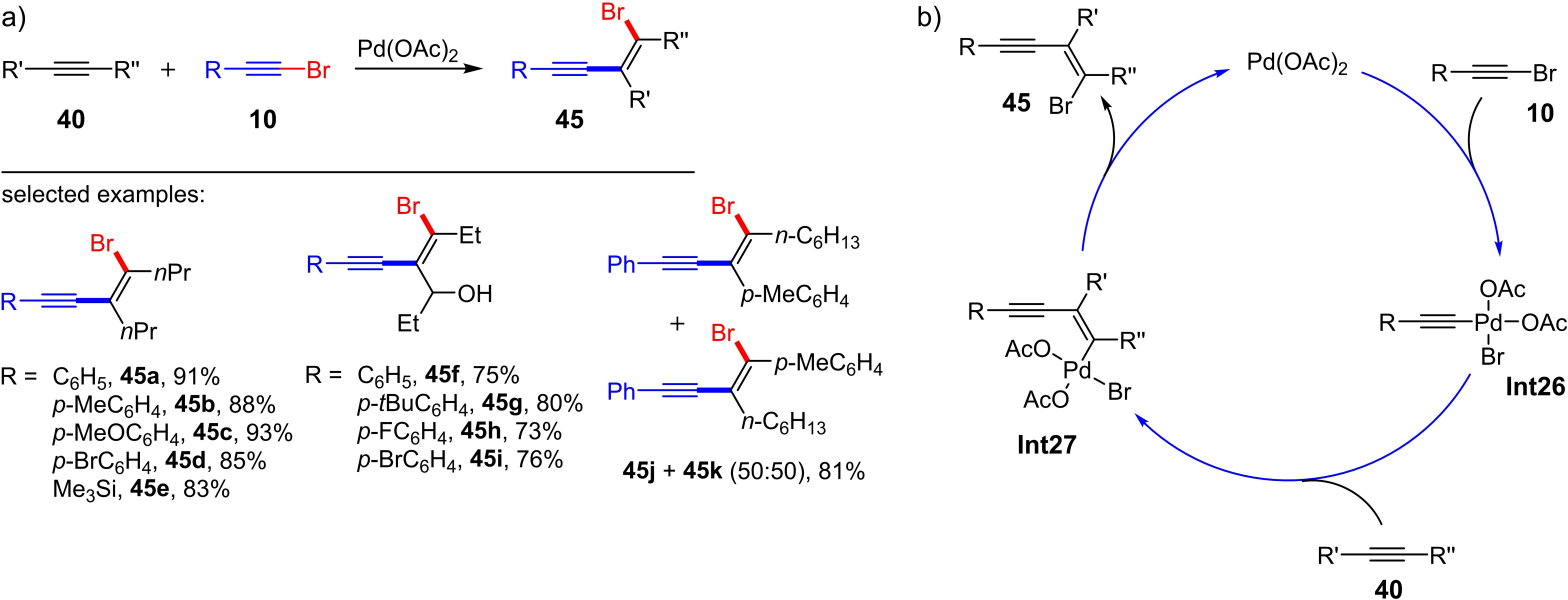
a) Palladium‐catalyzed bromoalkynylation of alkynes. Reaction conditions: Pd(OAc)_2_ (5 mol%), MeCN, room temperature. b) Proposed catalytic cycle for the palladium‐catalyzed bromoalkynylation of alkynes.^[28]^

One possibility for the direct synthesis of functionalized enynes is the addition of an activated alkyne to an alkyne. For this purpose, the alkynylstannylation,^[24]^ alkynylzirconation,^[25]^ alkynylboranation^[26]^ and alkynylcyanation^[27]^ of alkynes were developed in the early 2000s (Scheme 18a). The products of these carbometalation reactions can be further functionalized with various reagents. An important class of substances in these subsequent reactions are halogenated enynes (1‐halo‐1,3‐enynes **44**–**46**, Scheme 18b). The disadvantage of these methods is that stoichiometric amounts of a metal (Sn, Zr or B) and/or several reaction steps are generally necessary. Consequently, further studies have been conducted on developing metal‐catalyzed synthetic methods that lead to the halogenated enynes in a single step. In 2010, Jiang et al. presented the *cis*‐bromoalkynylation of alkynes for the first time.^[28]^ Here, the aryl‐ and alkyl‐substituted bromoacetylenes **10** (one example with an iodoalkyne) and the internal alkynes **40** were converted into the corresponding 1‐bromo‐1,3‐enynes **45** in the presence of catalytic amounts of Pd(OAc)_2_ (5 mol%) in acetonitrile at 35 °C (22 preparative examples with yields of up to 91 %; Scheme 19a).

A disadvantage of this Pd‐catalyzed method is the formation of both regioisomers when unsymmetrical substituted alkynes were used as starting materials. The conversion of an arylalkyl‐substituted alkyne takes place without regioselectivity so the conjugated and cross‐conjugated enynes (**45 k** and **45 j**) are formed in a 1 : 1 mixture (Scheme 19a). However, the Pd‐catalyzed bromoalkynylation of alkylalkynes bearing a hydroxy group in the α‐position (i. e. propargyl alcohols) leads to only one regioisomer (**45 f**–**45 i**, Scheme 19a) in good yields (approx. 80 %). The authors do not present an explanation for this selectivity, but one can speculate that the orientation of the alkyne is controlled by the coordination of the oxygen atom to the Pd metal center.

To investigate the reaction mechanism, the simultaneous reaction of haloalkynes (bromophenylacetylene **10 a** or iodophenylacetylene **21 a**) and 4‐octyne with stoichiometric amounts of PdCl_2_ was investigated.^[28]^ It was found that the halogen atoms in the halogenated main products originate from the haloalkynes. Jiang et al. concluded that the mechanism involves an unusual oxidative addition of the PdX_2_ species, rather than direct halopalladation of the alkyne. Based on these findings, the authors presented a plausible mechanism (Scheme 19b): The Pd‐catalyzed bromoalkynylation of alkynes is initiated by an oxidative addition of the Pd(II) salt to the bromoalkyne **10** and leads to the rare Pd(IV) species **Int26**. This is followed by the *cis*‐insertion of the alkyne **40** into the Pd−C bond resulting in the formation of the *cis*‐alkynylvinyl palladium species **Int27**. Finally, the reductive elimination takes place with release of the bromoalkynylation product **45** and the catalytically active Pd(II) species.

**Scheme 20:**
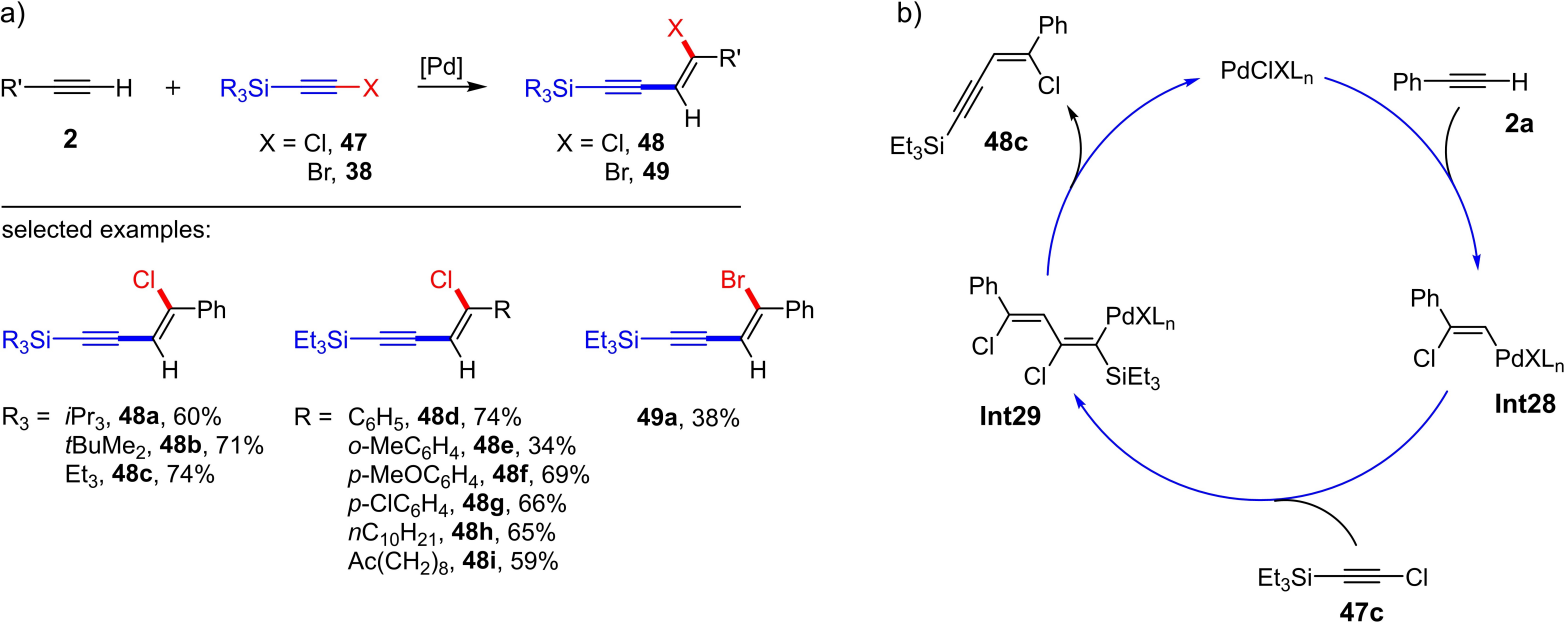
a) Palladium‐catalyzed addition of silyl‐substituted haloalkynes to terminal alkynes. Reaction conditions: [Pd_2_(dba)_3_], PPh_3_, decalin, 130 °C, 6 h. b) Possible reaction mechanism.^[29]^

A short time later, Oshima and Yorimitsu et al. described the Pd‐catalyzed addition of silyl‐substituted chloroalkynes **47** (one example with a bromoalkyne) to terminal alkyl‐ and arylalkynes **2** (Scheme 20a).^[29]^ The reaction takes place at a temperature of 130 °C in decalin in the presence of catalytic amounts of [Pd_2_(dba)_3_] and PPh_3_ and delivers the *cis*‐chloroalkynylation products **48** in yields of up to 74 % (13 examples) within six hours. The products of this chloroalkynylation reaction are particularly interesting as they can be further functionalized both via the halogen atom (e. g. via Suzuki reaction) and via the alkyne (by Sonogashira reaction) after deprotection of the silyl group.

To elucidate the reaction mechanism, the authors first examined a Pd(0)/Pd(II) catalysis cycle by selectively synthesizing a Pd(II) alkynyl compound and treating it with phenylacetylene in decalin for 6 h at 130 °C. The analysis revealed a complex mixture containing, among other by‐products, products of the trimerization of phenylacetylene. Thus, a Pd(0)/Pd(II) mechanism was unlikely. A Pd(II)‐based mechanism is therefore more realistic, in which an active chloropalladium(II) complex (PdClXL_*n*_) is involved in the *syn*‐chloropalladation of the terminal alkyne **2 a** and leads to the formation of the 2‐chloroalkenyl palladium species **Int28** (Scheme 20b). The insertion of the haloalkyne **47 c** into the Pd−C bond subsequently yields the dienyl palladium complex **Int29**. From the latter the *cis*‐chloroalkynylation product **48 c** and the catalytically active Pd(II) species are released by *anti*‐β‐chloride elimination.^[29]^

**Scheme 21:**
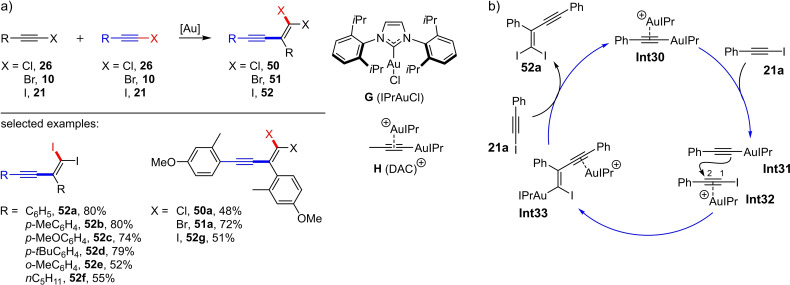
a) Dimerization of haloalkynes with dual‐activation catalysts. Reaction conditions: DAC⋅PF_6_^−^ or DAC⋅NTf_2_^−^, MeCN, 70 °C, 14 h. b) Mechanistic proposal for the dimerization with dual‐activation catalysts.^[29]^

In 2015, Hashmi et al. presented the first gold‐catalyzed haloalkynylation of an alkyne.^[30]^ In their seminal work, they converted aryl‐substituted iodoalkynes (**21**) at 70 °C in acetonitrile into the corresponding head‐to‐tail dimerization products **52** using dual‐activation catalysts (DAC; 5 mol%) (Scheme 21a). Interestingly, they were also able to successfully isolate a 1,1‐diiodinated enyne (**52 f**) starting from an alkyl‐substituted iodoalkyne. A total of eight preparative examples with yields of up to 80 % were presented; one example of the conversion of an aryl‐substituted bromo‐ and chloroacetylene, respectively, was also presented (Scheme 21a).

To investigate the reaction mechanism, they first checked whether the reaction proceeds via dual gold catalysis,[^31^, ^32^] i. e. a catalyzed reaction involving two gold centers.^[30]^ Therefore, they treated iodophenylacetylene (**21 a**) with stoichiometric amounts of IPrAuPh resulting in the formation of the gold acetylide **Int31** (Scheme 21b), which could be proved by means of ^1^H NMR spectroscopy. Accordingly, they assumed that **Int31** must play a key role in the mechanism and postulated the reaction cycle shown in Scheme 21b: The first step is the transfer of the [IPrAu] fragments to the haloalkyne **21 a** by the DAC to form **Int31**. In the next step, the triple bond of another iodoalkyne molecule **21 a** is activated by the cationic gold species; this leads to the formation of the π‐complex **Int32**. Subsequently, the C−C bond formation takes place through the nucleophilic attack of the σ‐activated gold acetylide **Int31** on the C2 atom of **Int32** resulting in the generation of the vinyl gold species **Int33**. In the last step, the catalyst is transferred to a new iodoalkyne molecule, so that the enyne **52 a** and the gold acetylide **Int30** are released.^[30]^ The gold(I)‐catalyzed haloalkynylation of alkynes presented by Hashmi was insofar groundbreaking as it was at that time one of the very few gold(I)‐catalyzed reactions in which the triple bond from the starting material reappears in the product; usually triple bonds in gold(I) catalysis are consumed during the reaction course.[^33^, ^34^] Considering the regioselectivity found for this Au(I)‐catalyzed haloalkynylation, the reaction can be described as addition of a halogen cation leading to a aryl‐stabilized carbenium ion, followed by addition of an alkynyl anion to an alkyne unit (synthon I see Scheme 1).

**Scheme 22:**
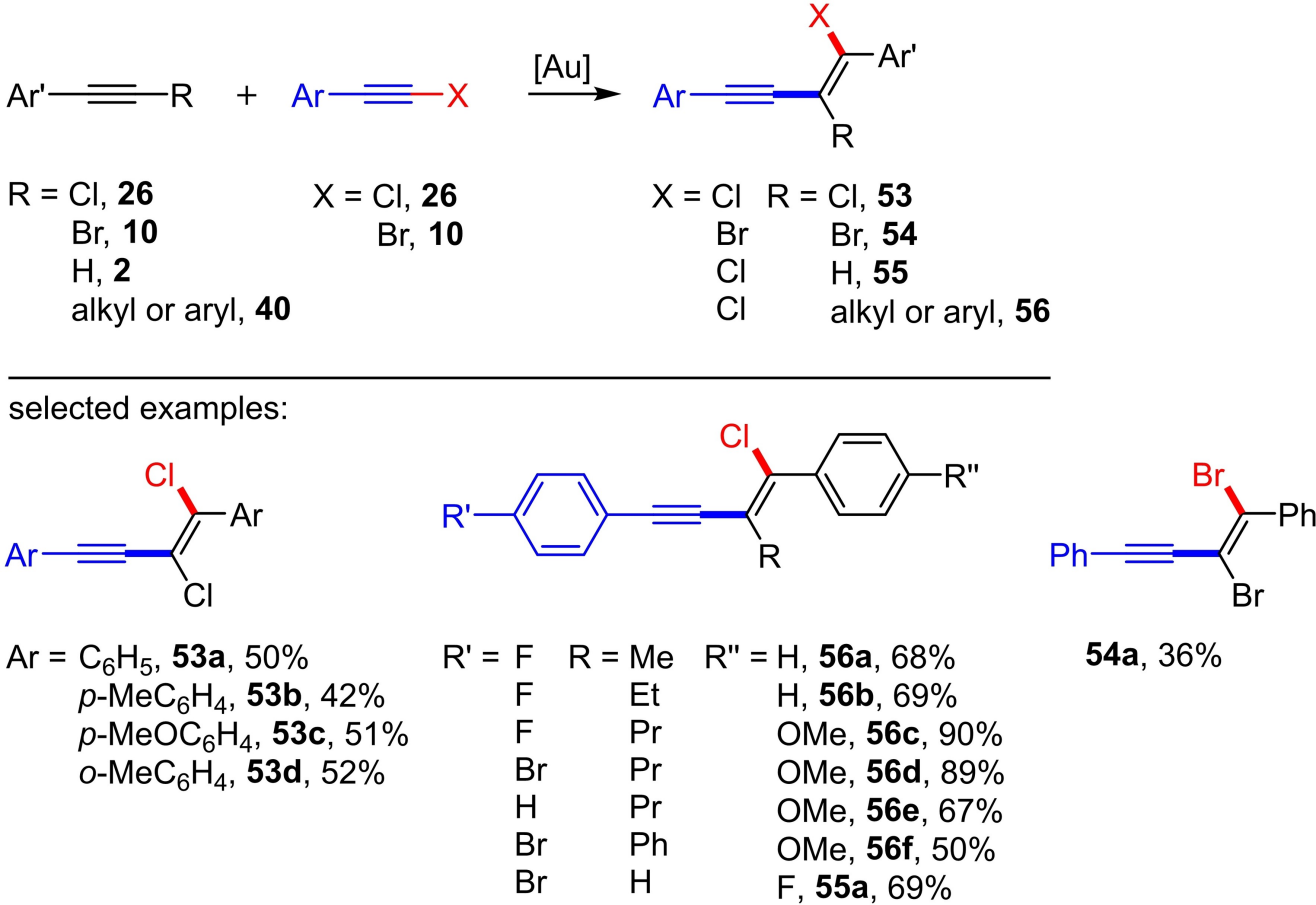
Gold(I)‐catalyzed haloalkynylation of aryl alkynes. Reaction conditions: [JohnPhosAu(NCMe)]SbF_6_ (5 mol%), DCM or DCE.[^35^, ^36^]

In 2019 Haberhauer et al. reported the successful head‐to‐head dimerization of chloro‐ and bromoalkynes (Scheme 22).^[35]^ This reaction represents a *cis*‐haloalkynylation of haloalkynes, which allows the construction of conjugated 1,2‐dihalogenated enynes (**53** and **54**) in just one synthetic step. The crystal structure analysis showed that the enyne **53** represents the *E* isomer. At that time, this structural motif has not been synthetically accessible and was only discussed as a possible by‐product in the thermal oligomerization of chloroacetylenes.^[37]^ However, the thermal dimerization of chloroacetylenes only yields inconsistent product mixtures.[^38^, ^39^, ^40^] The authors reaction protocol describes the treatment of the aryl‐substituted haloalkynes **10** and **26**,respectively, with the cationic gold complex [JohnPhosAu(NCMe)]SbF_6_ (5 mol%) in dichloromethane at room temperature. The isolated yields (up to 57 %) are moderate (7 examples; see Scheme 22).^[35]^

One year later, they were able to extend the gold(I)‐catalyzed *cis*‐haloalkynylation to arylalkyl alkynes and diaryl alkynes (**40**) as well as to terminal aryl alkynes (**2**) (Scheme 22).^[36]^ For this purpose, the aryl‐substituted chloro‐ or bromoalkynes (**10** or **26**) were reacted with the aryl alkynes (**2** or **40**) in 1,2‐dichloroethane (1,2‐DCE) at room temperature in the presence of [JohnPhosAu(NCMe)]SbF_6_. The special feature of this gold(I)‐catalyzed haloalkynylation of aryl alkynes is the regioselective synthesis of the corresponding conjugated enynes. In the case of the Pd‐catalyzed variant by Jiang et al. no regioselectivity was found for the aryl alkynes as starting material (see Scheme 19).^[28]^ The selective formation of the conjugated regioisomer in the gold(I)‐catalyzed variant can be explained by the stability of the intermediate cation. A total of 20 preparative examples with yields of up to 90 % were presented (Scheme 22). Interestingly, the *cis*‐chloroalkynylation of a thiophene‐substituted alkyne is also described, albeit with a low yield (24 %).^[36]^

**Scheme 23:**
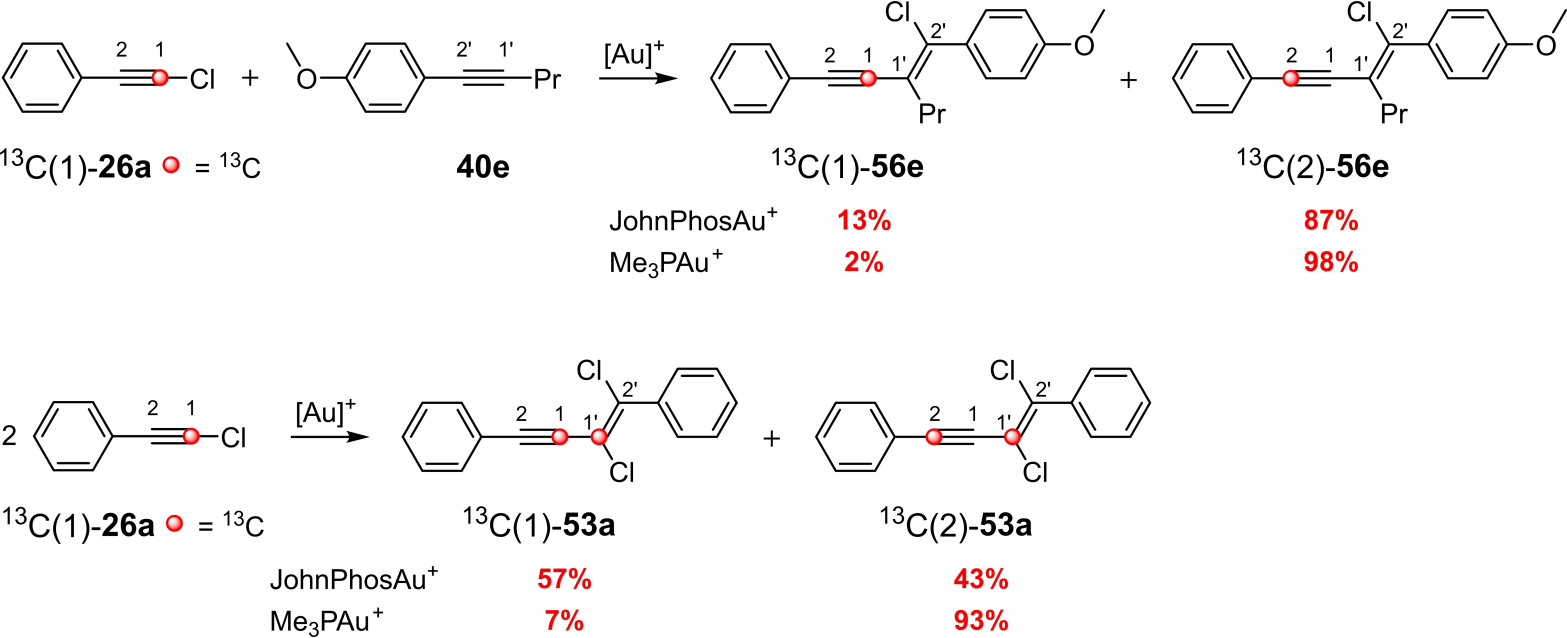
Gold(I)‐catalyzed 1,2‐haloalkynylation of aryl alkynes using ^13^C‐labeled starting materials and different phosphine ligands.^[41]^

Various routes have been discussed for the reaction mechanism of the gold(I)‐catalyzed haloalkynylation of alkynes.[^35^, ^36^] An important aspect was the fact that dual gold catalysis could be ruled out through preliminary experimental investigations.^[35]^ Another important cornerstone was the mechanistic studies by means of ^13^C labeling experiments.^[41]^ For example, the dimerization of **26 a** and the reaction of **26 a** with **40 e** each yields two *cis*‐chloroalkynylation products, which differ in the position of the ^13^C‐labeled carbon atom (Scheme 23). In the case of ^13^C(2)‐**53 a** and ^13^C(2)‐**56 a**, the ^13^C‐labeled atom changes its position relative to the phenyl ring, i. e., a rearrangement must have taken place. The ratio of the two ^13^C‐labeled products depends both on the starting materials and catalysts and ranges between 57 : 43 and 2 : 98.

**Scheme 24:**
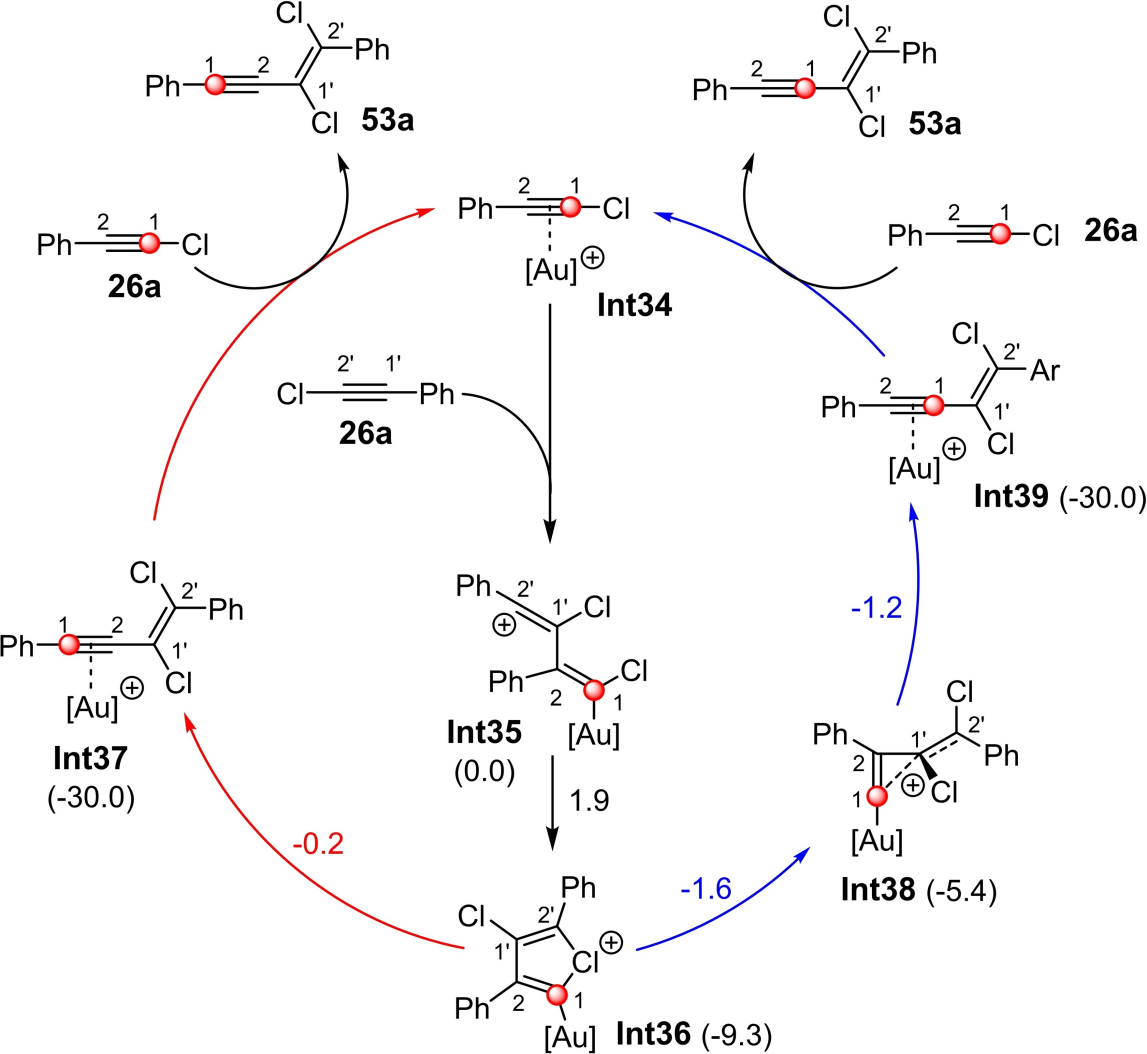
By means of B3LYP‐D3BJ(SMD) calculated catalytic cycle for the gold(I)‐catalyzed 1,2‐chloroalkynylation of chlorophenylacetylene (**26 a**). The free energies (ΔG) of the intermediates **Int36‐Int39** (numbers in brackets) and transition states (numbers above the arrows) are given in kcal/mol and are relative to **Int35**.^[41]^ [Au]^+^=JohnPhosAu^+^.

Extensive DFT calculations subsequently shed light on the mechanism and explained the product distribution found for ^13^C(1)‐**53 a** and ^13^C(2)‐**53 a** (Scheme 24).^[41]^ The first step is rate‐limiting and represents the nucleophilic attack of alkyne **26 a** on the C2 atom of the cationic gold(I) haloalkyne complex **Int34** yielding the vinyl cation **Int35**. The attack on the C1 atom of **Int34**, which has been also discussed,^[35]^ is 6 kcal/mol higher in energy and can therefore be ruled out as a possible reaction path. A rotation along the C1’‐C2 axis and the nucleophilic attack of the halogen atom on the C2’ atom yields the chloronium ion **Int36**. Starting from **Int36**, the product can be formed via two independent pathways (red and blue arrows in Scheme 24). While the opening of the chloronium ion **Int36** results in the generation of the gold(I) cyclopropenylmethyl cation **Int38** (blue arrow), the simultaneous opening of the chloronium ion **Int36** and 1,2‐aryl rearrangement leads directly to the gold(I) enyne complex **Int37** (red arrow).

According to the calculations, the activation barriers for these two steps are energetically similar (see Scheme 24); therefore, both paths can be passed through. The gold alkyne complex **Int39** can be formed from the cyclopropenylmethyl cation **Int38** via 1,2‐alkenyl rearrangement. In both cases, the catalytic cycle is completed by the transfer of the gold catalyst with release of the 1,3‐enyne **53 a**. The products of the two reaction pathways differ only in the position of the ^13^C‐labeled atoms. The catalyst‐related dependence of the experimentally found tendencies for the ratio ^13^C(1)‐**53 a** and ^13^C(2)‐**53 a** agrees very well with the calculations for the various catalyst systems. The cyclopropenylmethyl cation **Int38**, which occurs as intermediate in the catalytic cycle, represents an unusual and previously undiscovered gold(I) intermediate. Therefore, the authors investigated more intensively the electronic properties of this type of intermediate by means of quantum chemical calculations.^[41]^

If one compares the catalytic cycle (Scheme 24) of this reaction with that of the haloalkynylation of alkynes using dual‐activation catalysts (Scheme 21b), it becomes evident that these reactions have to be described by different synthons. While the addition of a halogen cation followed by an alkynyl anion is present with the dual‐activation catalysts (synthon I see Scheme 1), the addition using a simple cationic gold catalyst is best described by the addition of an alkynyl cation leading to an aryl‐stabilized carbenium ion, followed by the addition of a halide (synthon III).

**Scheme 25:**
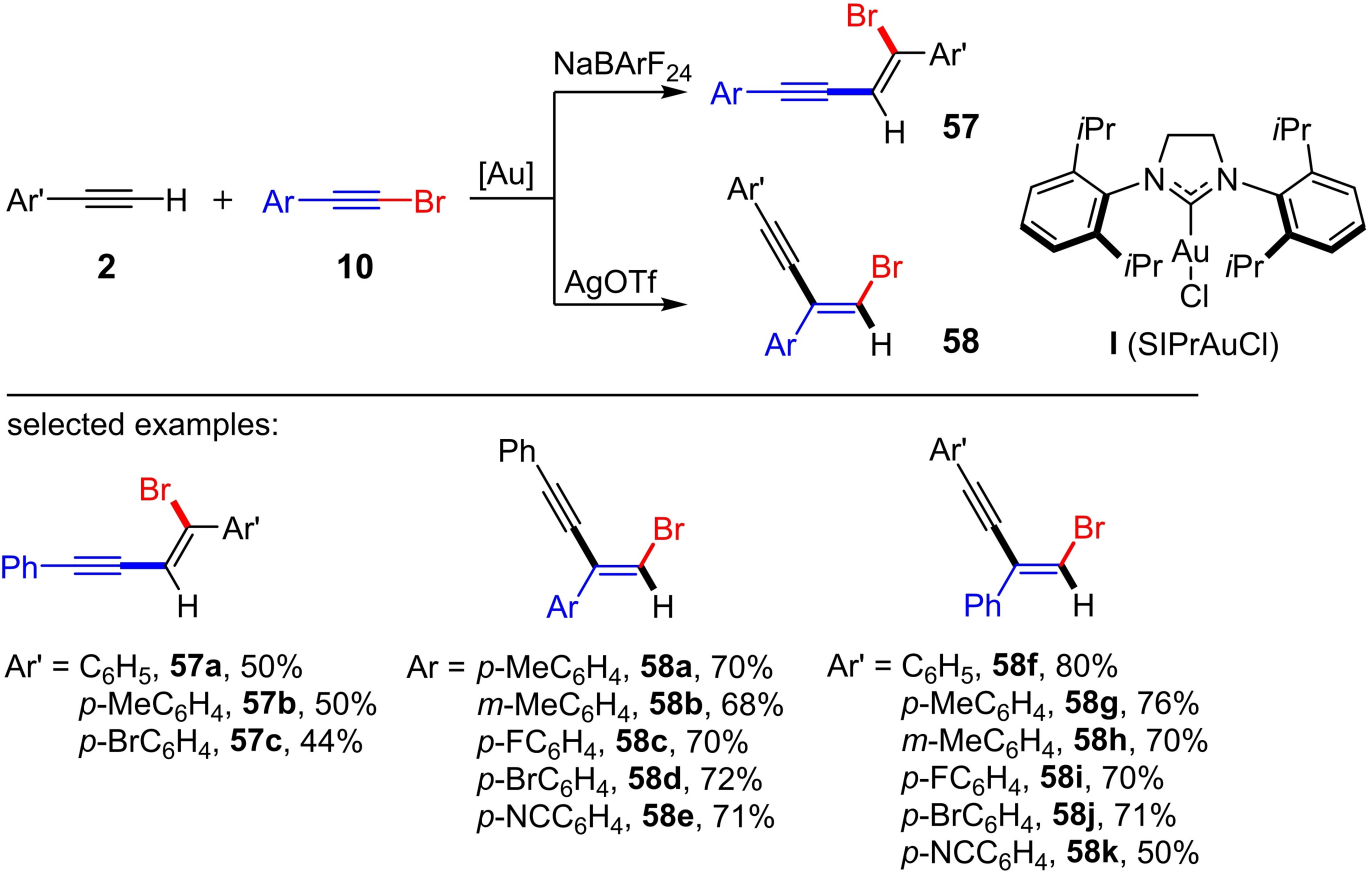
Au‐catalyzed bromoalkynylation of terminal alkynes and hydroalkynylation of aromatic bromoalkynes. Reaction conditions: **I** (SIPrAuCl) (5 mol%), NaBArF_24_ (5 mol%) or AgOTf (5 mol%), CHCl_3_, room temperature.^[42]^

Shortly thereafter, Fernández, Lassaletta et al. showed that the reaction of the bromoarylalkynes **10** and the terminal alkynes **2** with SIPrAuCl (5 mol%) leads to two different products depending on the counterion (BArF_24_^−^ vs. OTf^−^) (**57** vs. **58**; Scheme 25).^[42]^ While the reaction with BArF_24_^−^ affords the gold(I)‐catalyzed *cis*‐bromoalkynylation of the terminal alkyne **2**, the usage of OTf^−^ results in the *trans*‐hydroalkynylation of the bromoarylalkyne **10** (Scheme 25). The authors explained the switch in the mechanism on the basis of the basicity of the counterion. For the *cis*‐bromoalkynylation of the terminal alkynes **2** with BArF_24_^−^ as counterion, the mechanism via 1,2‐aryl or 1,2‐alkenyl rearrangement, shown in Scheme 24, can be assumed. The use of OTf^−^ as a counterion leads to a deprotonation of the terminal alkyne **2** in the transition state. Hence, no subsequent rearrangement occurs, but a protodesauration results in the generation of the *trans*‐hydroalkynylation product **58**.

**Scheme 26:**
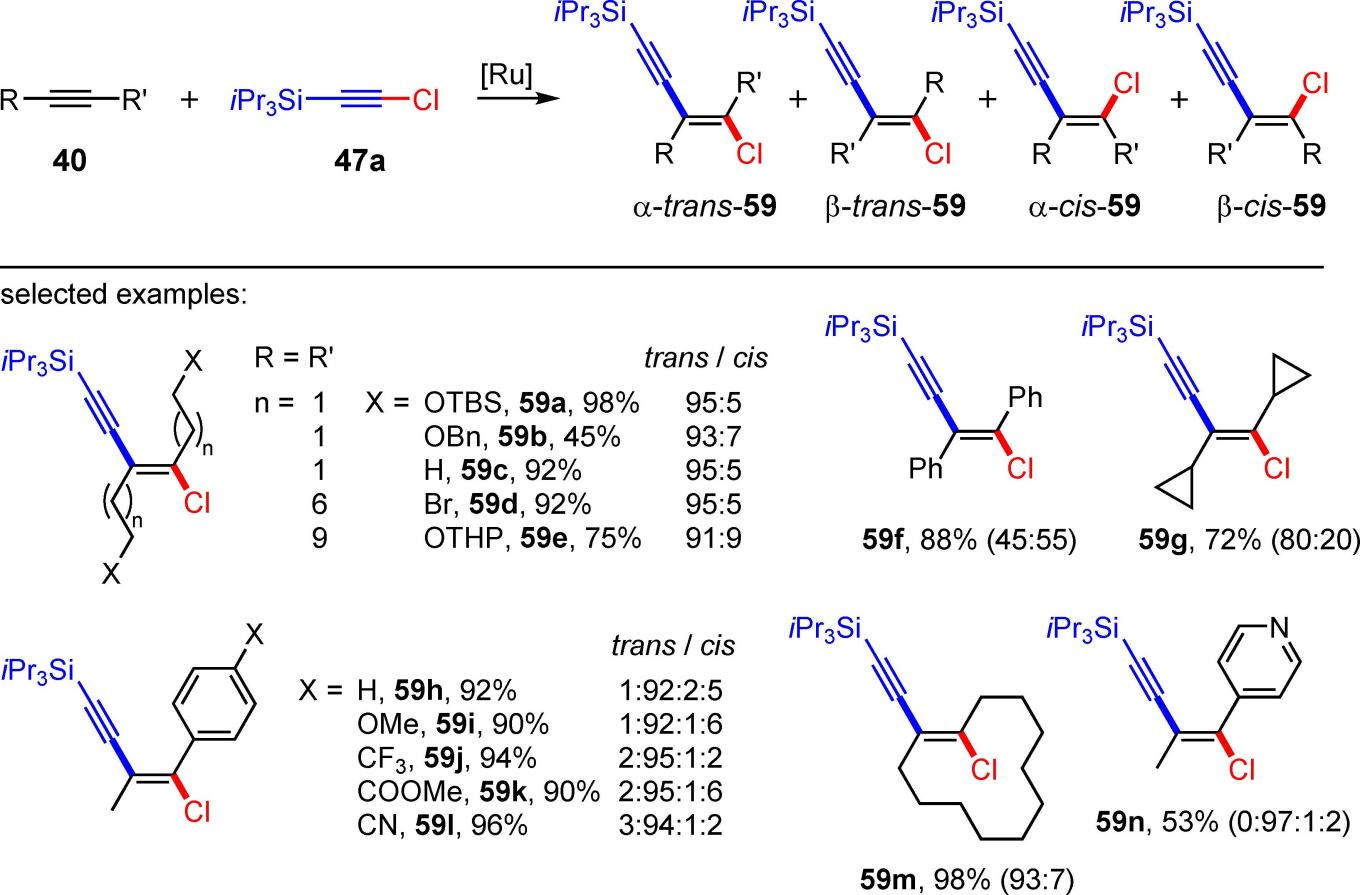
Ruthenium‐catalyzed *trans*‐chloroalkynylation of alkynes. Reaction conditions: [Cp*RuCl]_4_, 1,2‐dichloroethane, 80 °C.^[43]^

**Scheme 27:**
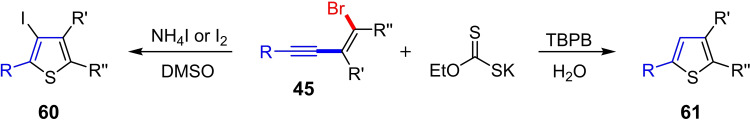
Chemoselective heterocyclization of bromoenynes **45** via a transition‐metal‐free sulfuration/cyclization process to the thiophenes **60** and **61**. TBPB=tetrabutylphosphonium bromide.^[49]^

Please note, from a stereochemical point of view the above discussed haloalkynylations using Pd and Au catalysts only lead to the *cis*‐addition products. The first *trans*‐haloalkynylation was recently presented by Fürstner et al.^[43]^ In this work, the authors describe the ruthenium‐catalyzed *trans*‐chloroalkynylation of internal alkynes with chlorotrialkylsilylacetylene **47 a** (Scheme 26). For example, the reaction of 3‐hexyne with **47 a** in 1,2‐DCE as solvent at 80 °C in the presence of [Cp*RuCl]_4_ (1.25 mol%, 12.2 mmol batch size) leads to the formation of the *trans*‐chloroalkynylation product **59 c** with a yield of 92 %. The observed *E*/*Z* ratio for **59 c** is higher than 95 : 5 (Scheme 26). The use of terminal tri(isopropyl)silylacetylenes affords the corresponding *trans*‐hydroalkynylation product.^[43]^ The scope of the reaction for other symmetrical alkynes was subsequently investigated. For alkyl‐substituted alkynes, yields of up to 98 % (*E*/*Z*=80 : 20 to 99 : 1) could be achieved (13 examples). The regioselectivity for the formation of the *trans*‐addition product decreases significantly only in the case of aryl‐substituted alkynes (**59 f**, *E*/*Z*=45 : 55). The reaction of chloroacetylene **47 a** with unsymmetrical alkynes leads, under the optimized reaction conditions, to the *trans*‐chloroalkynylation product **59**, whereby also other isomers, which are usually inseparable, are formed (α‐*trans*, α‐*cis* and β‐*cis*, see Scheme 26).^[43]^ Finally, by functionalizing their products, Fürstner et al. show how invaluable halogenated (chlorinated) enynes are for synthetic organic chemistry. Haloenyes are crucial precursors for the synthesis of highly substituted five‐ and six‐membered heterocyclic ring systems like furans,^[44]^ germoles,^[45]^ phospholenes,^[46]^ phospholes,^[47]^ siloles,^[48]^ thiophenes,^[49]^ quinolines^[50]^ and chromenes.^[51]^ One recent example uses the Pd‐catalyzed[^16^, ^28^] and the Au‐catalyzed bromoalkynylation^[36]^ of alkynes for the synthesis of bromoenynes **45**, which deliver highly substituted thiophenes (**60** and **61**) via a chemoselective heterocyclization (Scheme 27).^[49]^ Other examples include the application of haloenynes for the synthesis of cyclic enediynes^[52]^ and pentalenes.^[53]^

## 5 Conclusion

A variety of metal‐catalyzed haloalkynylation methods have been identified over the past decade to generate both C−X and C−C bonds. These protocols include the addition of haloalkynes to arynes, alkenes and alkynes. In the case of arynes, *ortho*‐haloalkynyl arenes are formed with copper being used as a catalyst. The haloalkynylation of alkenes has so far been conducted mainly via gold catalysis. From a mechanistical point of view, they all proceed with the same principle; namely the formation of the homopropargyl halides in which the halogen atom is bonded to the more substituted carbon atom. In this context, no change in regioselectivity has been achieved yet. The haloalkynylation of alkynes yields the corresponding halogen‐substituted enynes (1‐halo‐1,3‐enynes). Here, through a suitable choice of the catalytic system (Pd, Au & Ru), the regio‐ and stereoselectivity can be strongly influenced, so that currently almost all regio‐ and stereoisomers are accessible. Within the catalyst systems, the cationic Au catalysts lead to the highest regio‐ and stereoselectivities. However, their scope of application is still limited, since only aryl‐substituted alkynes can be used so far. This strict limitation does not apply to the Pd and Ru catalyst systems.

Future developments should expand the focus of the currently described reaction in the direction of regioselectivity and stereoselectivity. Moreover, the scope of application with regard to haloalkynes (especially alkyl‐substituted alkynes) and unsaturated systems should be broadened. Under these circumstances this reaction could become another important transformation of the routinely used C−C coupling reactions.

## Conflict of interest

There is no conflict of interest to declare.

6

## Biographical Information

*Dr. Mathis Kreuzahler studied technical chemistry at the Cologne University of Applied Sciences, where he received his B.Sc. in 2013. For his master's degree in chemistry, he changed to the University of Duisburg‐Essen and obtained his M.Sc. in 2017. Subsequently, he worked under the supervision of Prof. Dr. G. Haberhauer on the development of new gold‐catalyzed C−C coupling reactions (haloalkynylation reactions) and finished his doctorate in organic chemistry in 2021. After finishing his Ph.D., he joined Symrise AG as a laboratory manager, where he is working in the pilot plant on the process development of fine chemicals*.

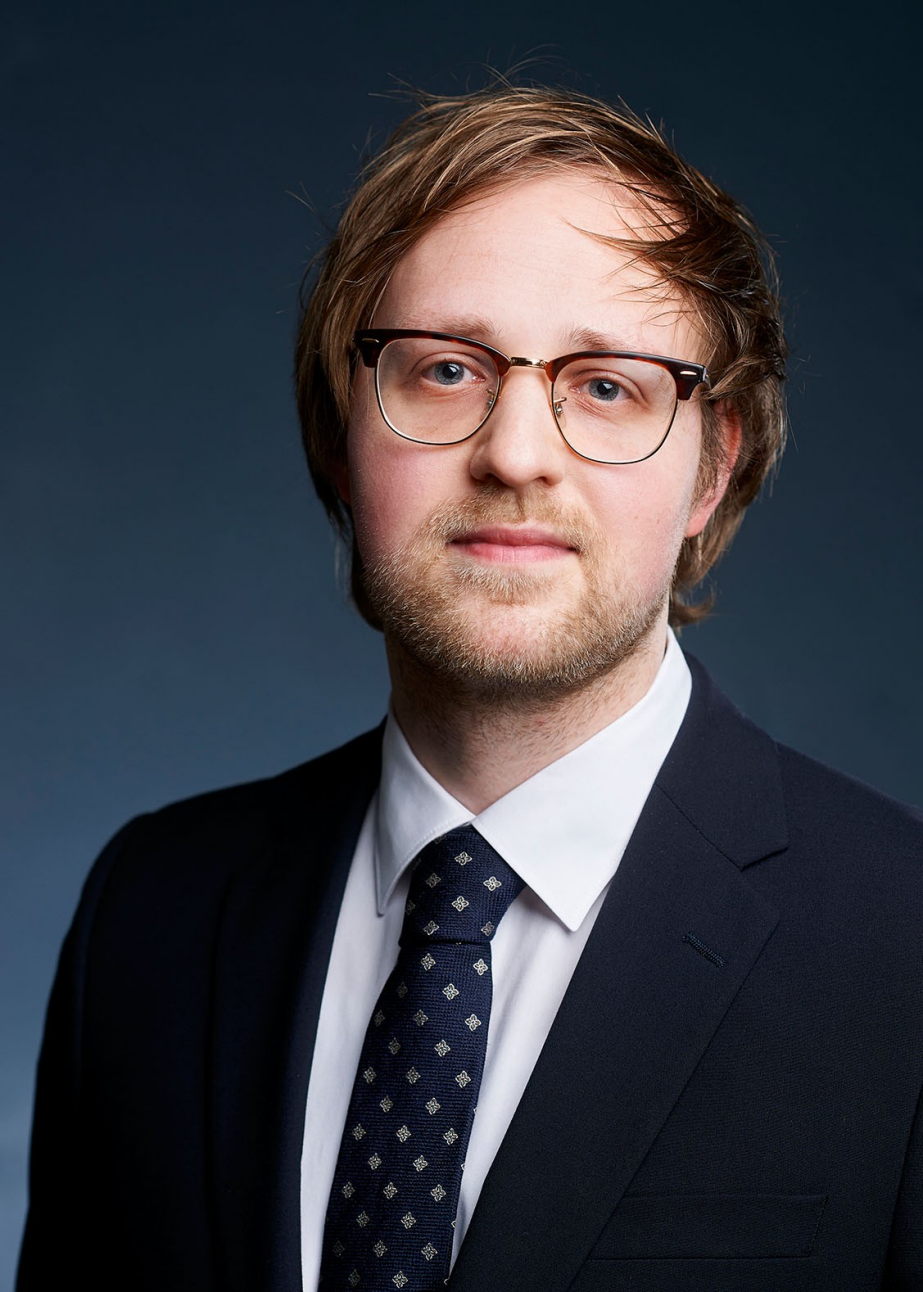

## Biographical Information

*Gebhard Haberhauer is Professor for Organic Chemistry at the University of Duisburg‐Essen. He studied chemistry at the universities of Vienna and Heidelberg and received his Ph.D. from the University of Heidelberg under the supervision of Prof. R. Gleiter. From 1999–2000 he was a postdoctoral fellow in the laboratory of J. Rebek, Jr. at The Scripps Research Institute in La Jolla. After working one year at BASF in Ludwigshafen, he returned to the University of Heidelberg, where he did his habilitation from 2001 to 2005. In 2005, he was appointed Professor for Organic Chemistry at the University of Duisburg‐Essen. His current research interests include chirality induction, synthesis of molecular switches and gold‐catalyzed haloalkynylation reactions*.

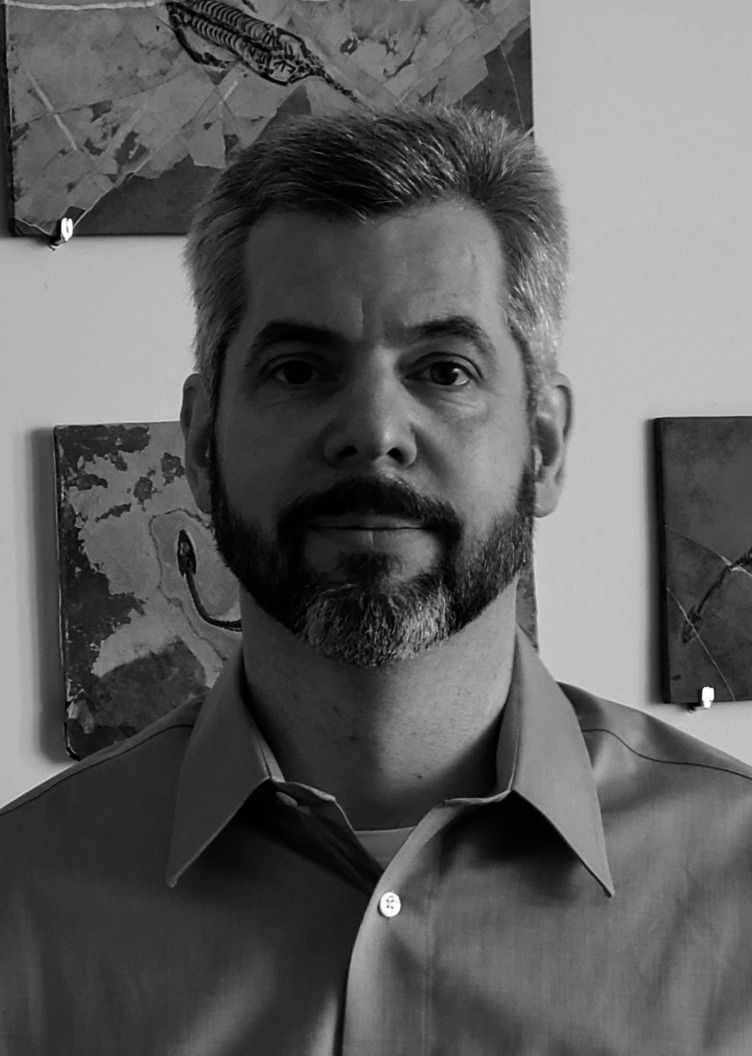

## Data Availability

Data sharing is not applicable to this article as no new data were created or analyzed in this study.
